# Solvent‐Mediated Dewetting Principles for Cell‐Sized Liposome Formation

**DOI:** 10.1002/smll.202512610

**Published:** 2026-02-01

**Authors:** Mostafa Bakouei, Tatiana Avsievich, Indraja Sundara Raju, David Stumpf, Ali Kalantarifard, Benny Ryplida, Caglar Elbuken

**Affiliations:** ^1^ Faculty of Biochemistry and Molecular Medicine University of Oulu Oulu Finland; ^2^ Faculty of Medicine University of Oulu Oulu Finland; ^3^ VTT Technical Research Centre of Finland Oulu Finland

**Keywords:** cell‐sized liposome, giant unilamellar vesicle, microfluidic liposome, solvent‐mediated dewetting, synthetic cell

## Abstract

Reconstitution of synthetic cells holds potential to advance synthetic biology, biomanufacturing, and therapeutics. Microfluidic generation of cell‐sized liposomes via double emulsion templating offers precise control over composition and formation process, yet the principles underlying solvent‐mediated dewetting remain poorly understood. Using a solvent combination of hexanol and paraffin oil, we demonstrate that solvent‐mediated dewetting liposome generation entails both solvent removal and the application of mechanical stimuli. Solvent removal suffices to induce the morphological transition from double emulsions to partially dewetted liposomes exhibiting low and high budding angles of the residual oil pockets. This transition is driven by relaxation of monolayer and membrane tensions, arising from the increased lipid packing density at the liposome interfaces during solvent depletion. While dewetting kinetics and intermediate stages are governed by solvent removal rate, complete dewetting is not spontaneous. Using optical tweezers, we identify tethering between the liposome and oil pocket and characterize the mechanical force required for liposome detachment. By integrating these principles, a predictive, high‐throughput approach for generating biocompatible, surfactant‐free liposomes is provided. These findings establish a mechanistic framework for liposome dewetting and, through similarities to lipid droplet morphogenesis, offer a protocell platform that could further the understanding of biological budding processes.

## Graphical abstract

Dynamics of solvent‐mediated dewetting for giant unilamellar liposome formation. Solvent removal, through changing lipid packing and membrane tension, is shown to induce partial dewetting into equilibrium low and high budding angle morphologies. Complete dewetting requires mechanical force, quantified using optical tweezers. The findings provide an understanding of dewetting‐based liposome formation and membrane‐oil droplet interactions.

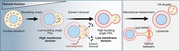

## 1 Introduction

Engineering artificial membranes and protocells through bottom‐up assembly has emerged as a transformative frontier in synthetic biology, with wide‐ranging implications in biomimetics [1, 2, 3], therapeutics [4, 5], and biomanufacturing [6, 7, 8]. Central to advancing this field is the reliable and reproducible production of giant unilamellar vesicles (GUVs) with precise structural and compositional control, achieved through the microfluidic generation of liposomes from double emulsion (DE) templates, which serve as a widely adopted benchmark for template‐based liposome formation [9, 10, 11, 12, 13]. Conversion of DE templates into fully formed liposomes relies on the dewetting transition. In the solvent‐mediated dewetting method, DE's oil shell consists of a mixture of good and poor lipid solvents, and the removal of the good lipid solvent drives oil retraction and enables lipid bilayer assembly [14, 15]. Previous studies advanced this method by enabling the generation of GUVs with customized configurations [16, 17, 18, 19], and in biocompatible systems [20, 21]. However, despite these advancements in GUV formation and application, the principles and mechanisms driving the dewetting transition remain to be elucidated.

Conventionally, the theoretical description of the dewetting transition relies solely on equilibrium thermodynamics, where spreading parameters of the DE phases, derived from interfacial tensions, predict the ultimate outcome of the dewetting either as a partially dewetted liposome (PDL) or a completely dewetted liposome (CDL) [15, 17, 22]. However, this approach does not capture the dynamics of the mechanisms governing dewetting or explain the nature of this transition, resulting in discrepancies between theoretical predictions and experimental outcomes. The current literature fails to address the existence of the intermediate morphological stages within the dewetting process, the possibility of achieving surfactant‐free liposome formation in the solvent‐mediated dewetting [17, 23], and the existence of an energy barrier that prevents spontaneous complete dewetting [24, 25, 26, 27, 28].

**FIGURE 1:**
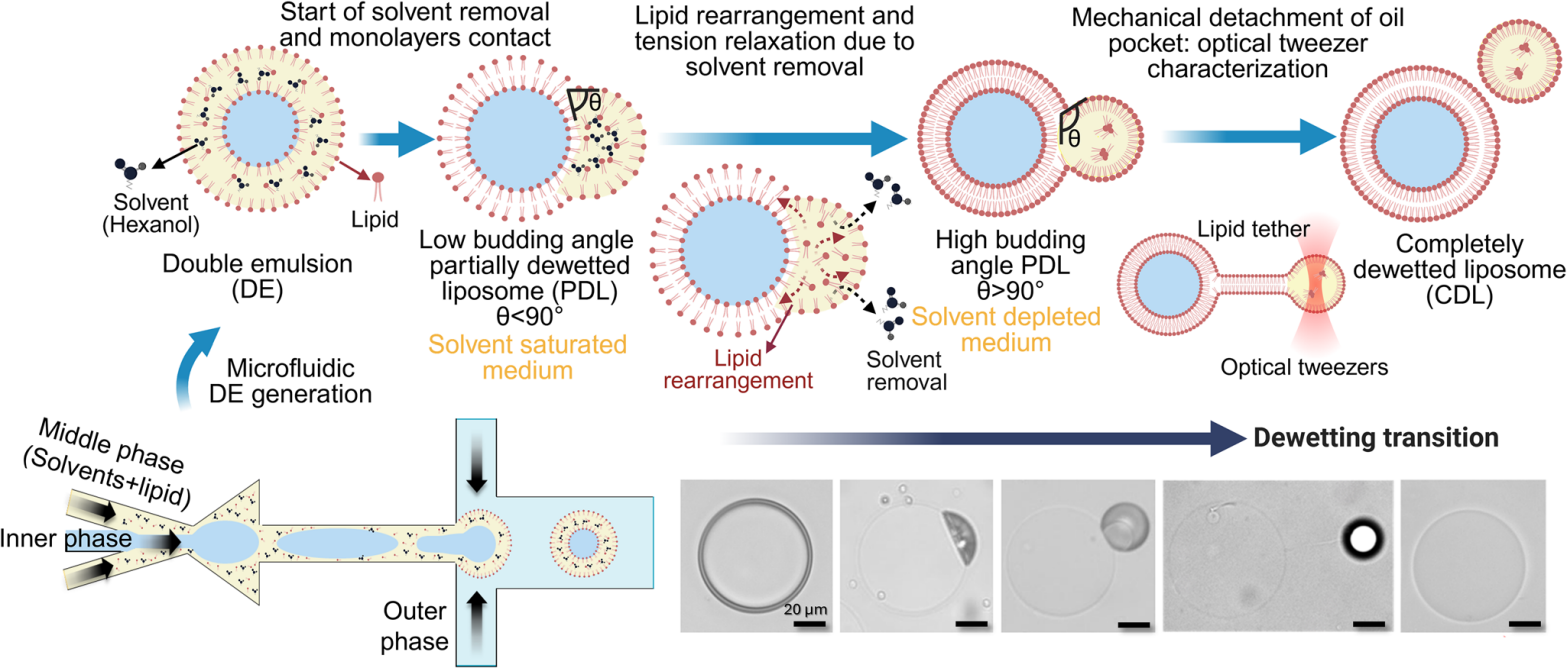
Illustration of the mechanisms governing the dewetting transition to generate liposomes from double emulsion templates. Dewetting of microfluidic formed DEs yields low budding angle PDL morphology in a solvent‐saturated medium. Continuous solvent removal drives the morphological transformation to high budding angle PDL. The final transition to a completely dewetted liposome is a non‐spontaneous step requiring external force quantified by optical tweezers.

In this work, we establish the principles of the solvent‐mediated dewetting process by determining the mechanisms involved and identifying the key physical factors through the quantitative characterization of the dewetting transition. Solvent‐mediated dewetting in our system is shown to be a dynamically complex physical phenomenon that requires lipid solvent removal and overcoming an energy barrier using external force. We reveal that solvent removal not only triggers the two monolayers to contact [17, 22], but that its depletion rate defines the kinetics of dewetting and the morphological progression of PDL from low budding angle (<90° oil droplet contact angle) to high budding angle (>90° angle). Furthermore, we demonstrate that hexanol depletion dictates lipid rearrangement and affects lipid monolayers and bilayer packing. This dynamic reorganization reduces interfacial tensions, thereby modulating the spreading parameters, favoring dewetting. Such dewetting by tension relaxation mimics lipid droplet budding from the endoplasmic reticulum membrane in biological cells [29, 30], suggesting a shared physical basis between synthetic and biological budding processes. While solvent removal leads to almost fully dewetted liposomes, complete separation of liposomes does not occur spontaneously. We use optical tweezers to quantify the mechanical forces required to detach the oil droplet and liposome, clarifying the nature of residual adhesion and the magnitude of the required force at the single liposome level. Figure 1 represents an overview of the dewetting process, highlighting the dewetting stages and the underlying mechanisms revealed in this study.

Together with our recently introduced hybrid (PDMS/glass capillary) microfluidic device [31] and the dewetting principles established in this study, we were able to achieve high‐throughput formation of GUVs. Furthermore, the selected solvent combination enables the formation of surfactant‐free biocompatible liposomes in a PDMS‐compatible system, enabling widespread application in biomembrane studies and synthetic cell research. The insights and design principles presented here elevate the fundamental understanding of membrane assembly processes, pave the way for the application of microfluidic liposomes in synthetic cell research, and help in the mechanistic understanding of biological budding processes such as lipid droplet budding from the endoplasmic reticulum [32, 33], vesicular organelle fission [34], crypt budding [35], and membrane budding [36, 37, 38].

## 2 Results

### 2.1 Solvent Removal Regulates Dewetting Dynamics

**FIGURE 2:**
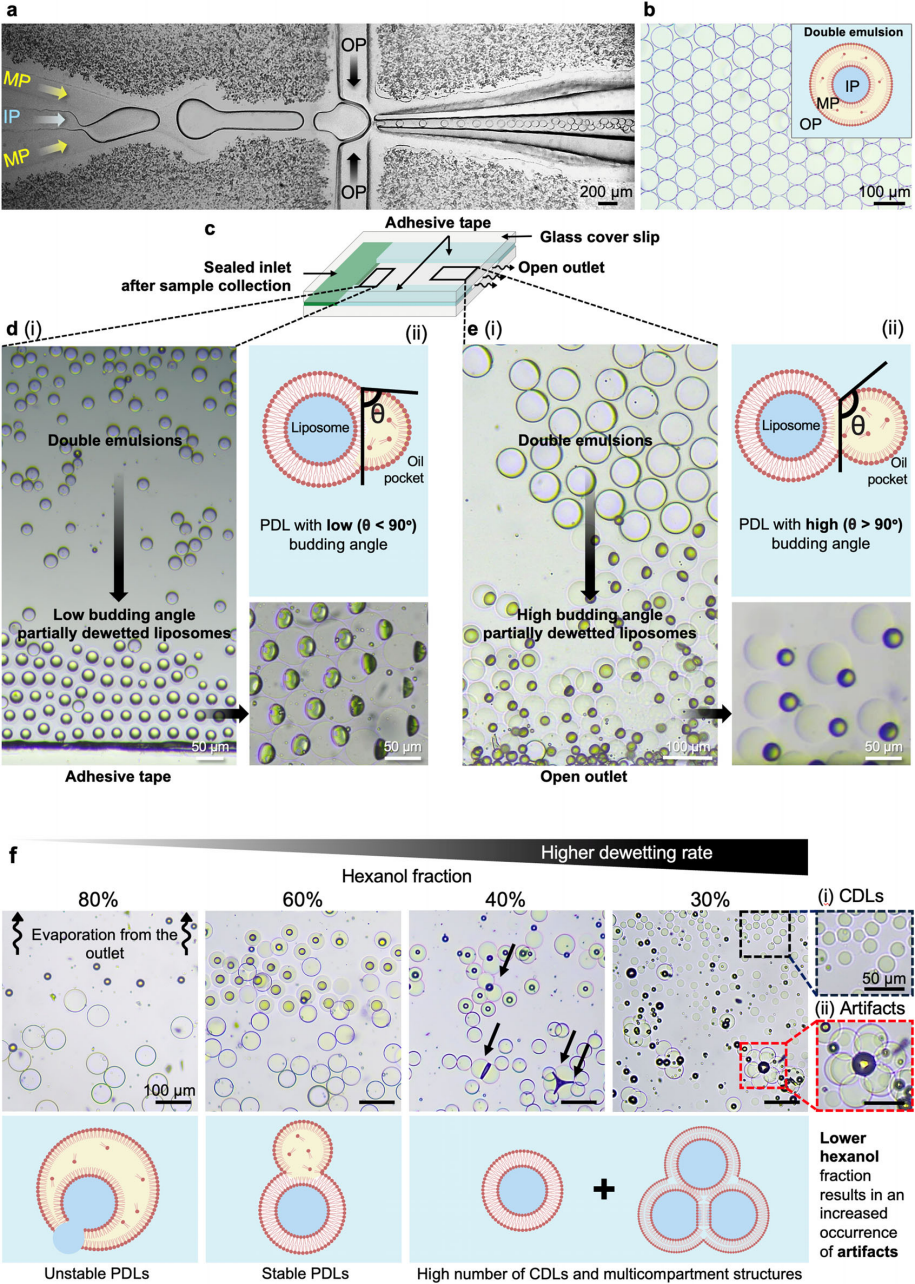
Spatially non‐uniform partial dewetting transition reveals the regulatory role of solvent removal. (a) Formation of DEs using a hybrid glass capillary‐PDMS microfluidic chip, depicting the inner aqueous phase (IP), lipid‐oil middle phase (MP), and outer aqueous phase (OP). (b) Microscopic image of ultra‐thin shell DEs collected into a glass chamber, with an inset schematic of their structure. (c) Schematic of the glass chamber with adhesive (semipermeable) tape walls, sealed inlet after DE collection, and open (permeable) outlet. (d‐i) Dewetting gradient observed near the semipermeable adhesive tape: intact DEs in the central region and partially dewetted liposomes (PDLs) with a low budding angle (*θ* < 90°) near the semipermeable edges. (ii) Side‐view and schematic show low budding angle PDLs. (e‐i) Dewetting gradient near the open outlet: DEs rapidly transition into PDLs with high budding angle. (ii) Side‐view image and schematic demonstrate high budding angle (*θ* > 90°) PDL morphology. (f) Microscopy images show double emulsions (DEs) with varying hexanol fractions in MP (80%, 60%, 40%, 30%) after 5 min in open glass chambers, with corresponding schematics illustrating dewetting outcomes observed for each concentration. Lower hexanol fraction accelerates dewetting and increases CDL yield, but also introduces formation artifacts (multicompartment structures) shown in the insets for 30% fraction.

DE templates were prepared using a recently introduced hybrid, surface treatment‐free microfluidic platform consisting of a PDMS chip integrated with a glass capillary (Figure 2a) [31]. Three phases were supplied into the microfluidic device to form DEs: (i) an aqueous inner phase (IP), (ii) lipids in a mixture of 60% hexanol and 40% paraffin oil as the two solvents in the middle phase (MP) chosen as a biocompatible and PDMS friendly alternative to the chloroform‐hexane solvent combination [21], and (iii) an aqueous outer phase (OP). The microfluidic system was operated in double dripping mode, enabling high‐yield formation of thin‐shell DEs (Figure 2a and Movie S1). The DEs were subsequently collected into a thin glass sample chamber (height: 120 µm) via capillary action. This chamber design facilitated the arrangement of DEs in a single layer, where DE diameter ranged between 60‐80 µm (Figure 2b). After DE collection, the inlet was sealed while the outlet remained open (Figure 2c). In Figure 2d‐i, DEs away from the adhesive semipermeable tape predominantly remained intact, while dewetting occurred near the tape within 3 h. This observation suggests that the gas permeability of the adhesive tape walls [39], plays a pivotal role in facilitating the dewetting transition. At dewetting sites, the lipid monolayers zip together upon contact to form a bilayer, and the middle phase wraps into an oil pocket attached to the liposome, yielding a PDL (Movie S2). The extent of dewetting was characterized by the oil pocket budding angle, *θ*, defined as the contact angle of the oil pocket. Figure 2d‐ii depicts the side view of PDLs with low budding angle (*θ *< 90°) near the sealed edges with a corresponding schematic. When both the inlet and outlet were sealed, dewetting proceeded extremely slowly, with the budding angle remaining consistently low. Complete dewetting did not occur even after 3 days (Figure S1). In contrast, when the outlet was left unsealed, a clear dewetting gradient emerged near the open outlet: DEs located further from the open end remained intact, whereas those closer transitioned into low‐budding‐angle PDLs. Budding angle of DEs at the air‐exposed outlet increased dramatically within 10 min, resulting in high budding angle morphology (*θ *> 90°) (Figure 2e). This spatially dependent dewetting behavior is attributed to the differing properties of the two solvents and their dynamic concentration changes in the middle and outer phases. Unlike paraffin oil, hexanol offers greater lipid solubility, is soluble in the aqueous phase, and exhibits moderate volatility (Table S1). To approximate the partitioning of hexanol between the MP and the aqueous phases, we used the partition coefficient of hexanol between hexadecane and water (ln *K* ≈ 1.23) [40], corresponding to 3.42 times higher concentration of hexanol in the MP than in the aqueous phase at equilibrium (Note S1). Given an initial 60% v/v of hexanol in the MP, the estimated equilibrium concentration in the aqueous phase reaches ∼ 144 g L^−^^1^, which exceeds the solubility limit of hexanol in water (∼ 5.9 g L^−^^1^). Consequently, the aqueous phase rapidly saturates with hexanol, limiting further partitioning unless hexanol is removed, e.g., by evaporation. In an open air‐exposed chamber, evaporation lowers the hexanol concentration in the OP, enabling continued diffusion from the MP and promoting further dewetting toward high‐budding‐angle liposomes (Figure 2e‐ii).

Furthermore, the effect of lipid solvent (hexanol) on dewetting dynamics was examined by varying its fraction (from 80 to 30%) in the MP before DE formation (Figure 2f). It is shown that decreasing the hexanol fraction indeed promoted a faster dewetting process since less solvent removal is required for dewetting; however, it also impaired DE formation (see Note S2 for details on solvent fraction effect).

**FIGURE 3:**
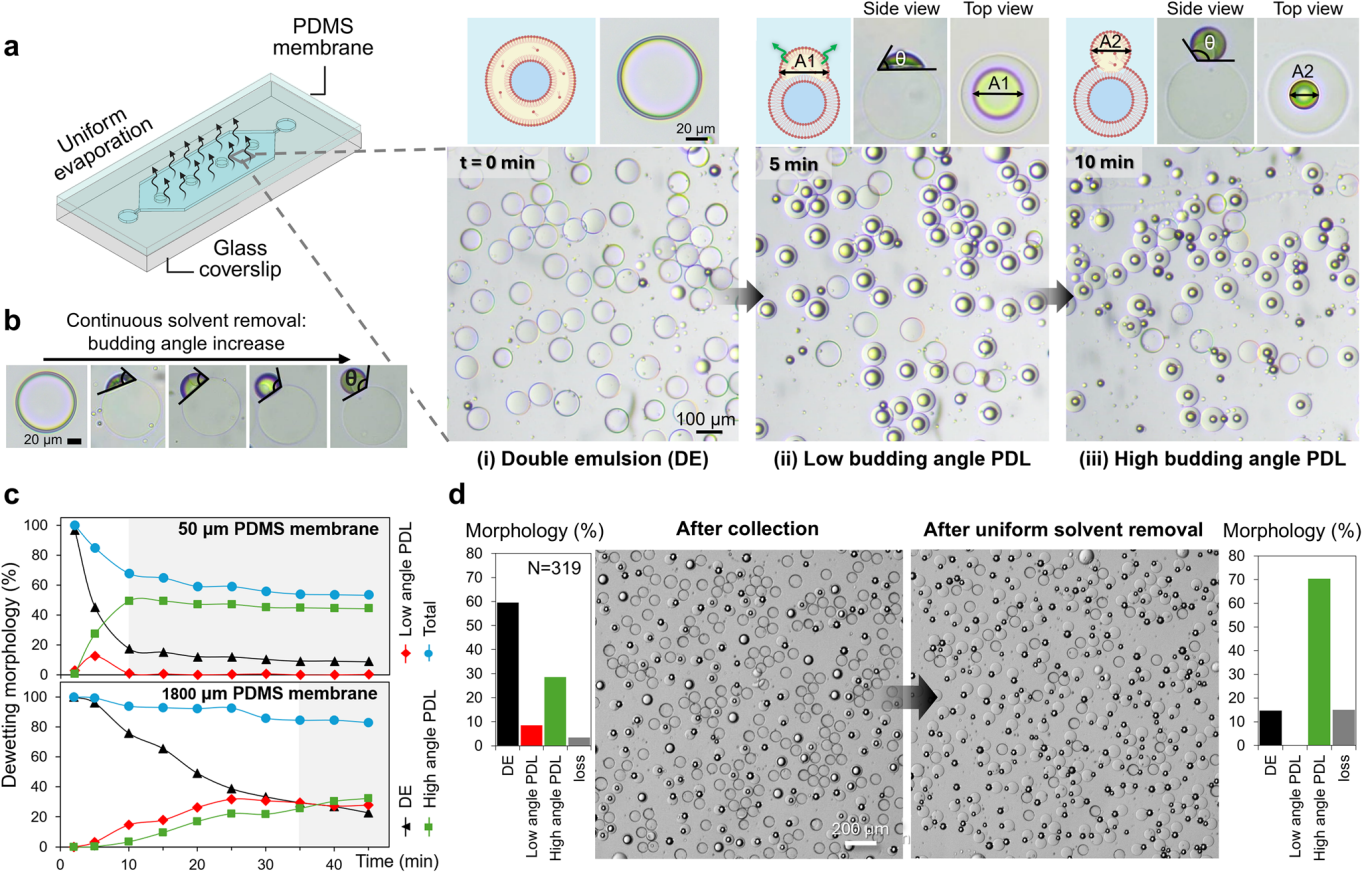
Regulation of partial dewetting transition under spatially uniform solvent removal. (a) Freshly collected DEs sequentially transition into low‐budding‐angle PDLs, and then, upon sufficient solvent removal, to high budding angle PDLs. The top panel shows the schematic, side‐view, and top‐views of dewetting morphologies at each stage. The reduction of the oil pocket area, from A1 to A2, in the top‐view corresponds to the transition from low to high budding angle in the side‐view. In the left panel, a schematic illustrates the gas‐permeable PDMS chamber. (b) Side‐view image series showing a gradual increase in budding angle as a function of continuous solvent removal from the oil pocket. (c) Dewetting transition dynamics in chambers with 50 µm and 1800 µm thick PDMS membrane (*N* = 450 DEs in each sample with a concentration of 45 DEs/mm^2^ and average DE shell thickness of 2 µm), showing the proportion of DEs (black triangles), low budding angle PDLs (red diamonds), high budding angle PDLs (green squares), and the total ratio of DEs and PDLs (blue circles) as a function of dewetting time. The gray zones indicate the time period at which the dewetting progression levels off. (d) Synchronized high yield dewetting transition in the 500 µm‐thick PDMS chamber with corresponding ratio of dewetting morphologies before and after uniform solvent removal (*N* = 319). The loss indicates ruptured DEs and PDLs.

To address the spatial non‐uniformity in the dewetting process observed in the glass chamber, the dewetting was examined under uniform solvent removal. For this purpose, the chamber containing DEs was sealed with a gas‐permeable PDMS membrane. This configuration ensured continuous and homogeneous evaporation of hexanol from the OP, promoting progressive diffusion of hexanol from the oil pocket. As shown in Figure 3a and Movie S3, this resulted in spatially synchronous dewetting within 10 min. The process is initiated by oil accumulation on one side of the DEs, followed by pocket formation with a low budding angle, and ultimately progresses to a high budding angle under continued hexanol removal (Figure 3a,b). The uniform and rapid dewetting in the gas‐permeable chamber highlights hexanol removal as the key driver of the morphological transition to the high budding angle state.

To examine the impact of solvent removal rate on the kinetics of the dewetting process, two gas‐permeable chambers with different permeabilities were investigated (Figure 3c). We compared the dewetting process in 50 µm and 1800 µm thick PDMS membranes with hexanol evaporation rates of 1.07 ± 0.11 µL min−^1^ cm^−^^2^ and 0.028 ± 0.007 µL min^−^^1^ cm^−^^2^, respectively. Similar initial concentrations and sizes of DEs were considered in both chambers, as the kinetics of the dewetting process are influenced by the initial solvent volume in the chamber. In the 50 µm PDMS membrane case, the significantly higher solvent removal rate triggered rapid dewetting, reaching equilibrium within the first 10 min, while in the 1800 µm membrane, it required approximately 35 min. Furthermore, the rapid solvent removal through the thin PDMS membrane caused the direct transition of most DEs to the high budding angle PDL, bypassing the low budding angle stage. This suggests that by the time dewetting begins, the fraction of hexanol in the oil pocket is sufficiently low to favor the immediate formation of high budding angle PDLs. Nonetheless, the rapid transition from DE to PDL in the 50 µm PDMS membrane chamber led to abrupt morphological changes, causing instabilities and resulting in a 44% loss compared to 17% in the 1800 µm membrane. As shown in Figure 3d and Figure S2, the application of a gas‐permeable chamber enables the full progression of solvent‐mediated morphological transition, resulting in high yield and uniform formation of high budding angle PDLs across the entire sample.

### 2.2 Origin of PDL Morphological Transformation upon Solvent Removal

**FIGURE 4:**
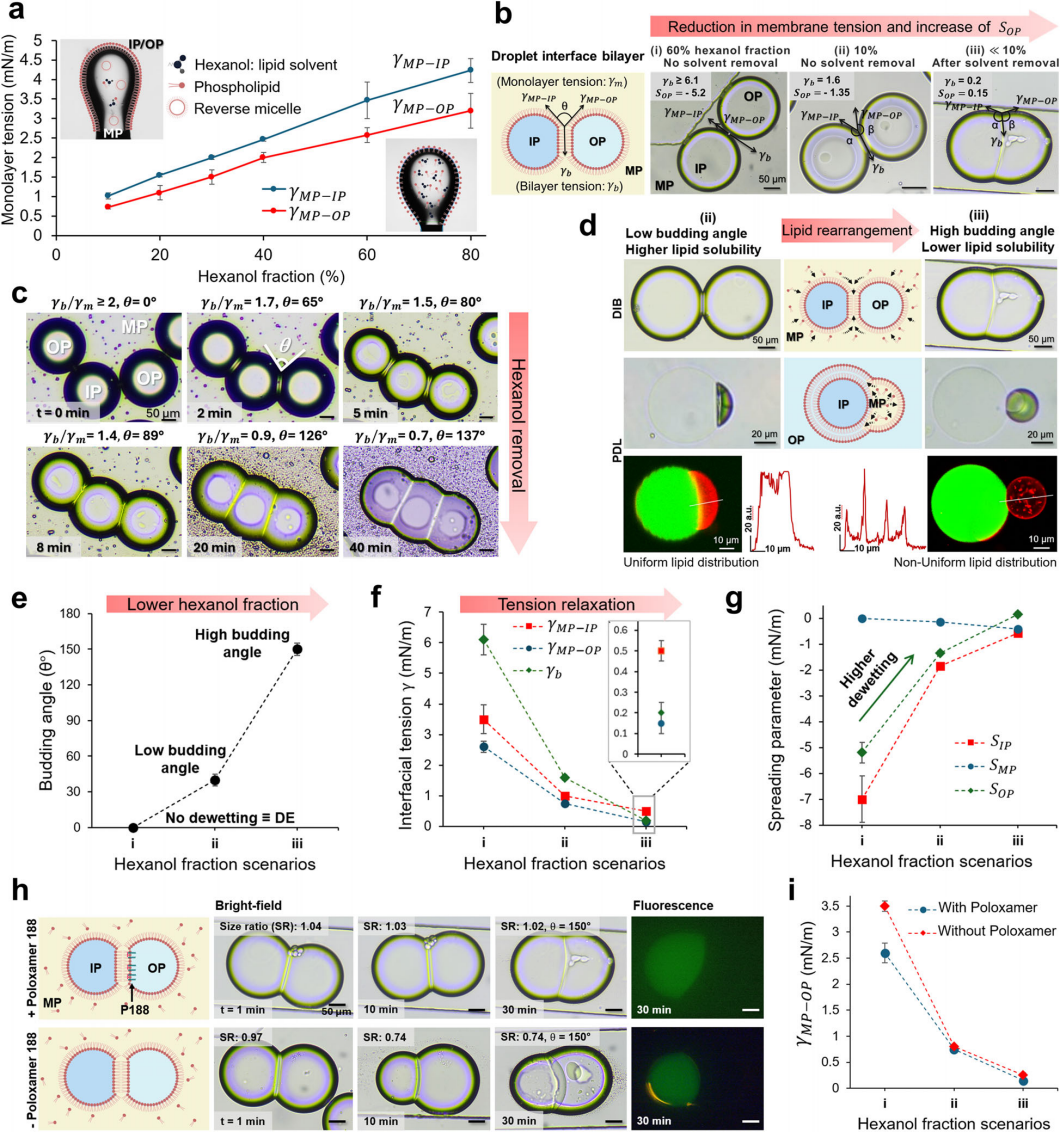
Reduction of monolayer and membrane tensions due to lipid rearrangement drives morphological transitions upon solvent removal. (a) Monolayer tensions at the MP‐IP and MP‐OP interfaces as a function of hexanol fraction. Schematics (inset) illustrate lipid distribution in pendant MP drops at low and high hexanol fractions. Error bars indicate mean ± SD (*N* = 3). (b) Schematic of a droplet interface bilayer (DIB) system and Equilibrium morphologies of DIBs under three fractions of hexanol in the continous MP: (i) 60% (no solvent removal), (ii) 10%; chip in a vapor‐saturated chamber preventing solvent removal, and (iii) ≪ 10%; a chip with initial MP containing 10% hexanol, allowed solvent removal to equilibrium (c) representative time‐lapse images showing budding angle increase as hexanol evaporates from the MP starting from initial 10% hexanol. (d) Equivalent morphological transitions (increase of budding angle) in DIB and PDL systems upon hexanol removal, schematic indicating potential directions for lipid redistribution toward monolayers and bilayer. The fluorescent images and corresponding intensity profile show Rhodamine‐stained lipid distribution at each morphology. (e) Budding angles under hexanol fraction scenarios (i)–(iii) shown in (b). Error bars indicate ±5° measurement tolerance. (f) Monolayer tensions (γ_MP − IP_, red square), (γ_MP − OP_, blue circle) and membrane bilayer tension (γ_b_, green diamonds), and (g) corresponding spreading parameters (S_IP_, red squares), (S_MP_, blue circles) and membrane bilayer (S_OP_, green diamonds) for conditions (i)–(iii). Error bars in (f) and (g) indicate the mean ± SD (*N* = 3). (h) Time‐lapse images of DIBs morphology upon hexanol removal in the presence and absence of poloxamer 188 show similar changes and equilibrium budding angle. A Significant reduction of droplet size ratio (SR), the ratio between diameters of DIB droplets, is observed in the absence of poloxamer. Fluorescent image (right) shows the high budding angle DIB with Alexa Fluor 488 stained IP; the OP remains unstained. (i) Middle phase‐outer phase monolayer tension (γ_MP − OP_) in the absence (red diamonds) and presence of poloxamer 188 (blue circle). Error bars indicate the mean ± SD (*N* = 3).

To elucidate the underlying mechanisms driving the morphological transition of PDLs from low to high budding angle upon hexanol removal, we analyzed lipid redistribution and interfacial tensions. Given that hexanol removal reduces its fraction in the oil pocket, we evaluated monolayer and membrane tensions in systems with varying hexanol fractions. Interfacial tension (**γ**) measurements reveal that decreasing the hexanol fraction in MP reduces the monolayer tension at both the middle phase‐inner phase (γ_*MP* − *IP*_) and the middle phase‐outer phase (γ_*MP* − *OP*_) interfaces (Figure 4a). Interestingly, this trend is opposite to that of a control system absent of lipids and surfactants, where paraffin oil, as a non‐polar oil, exhibits significantly greater interfacial tension than hexanol ($\gamma_{paraffin\;oil}∼\;42.5\;\mathrm{mN}\;m^{-1}\;\gg \;\gamma_{Hexanol}∼\;6.8\;\mathrm{mN}\;m^{-1})$, (Figure S3a). In the presence of lipids and surfactants, at high hexanol fractions, lipids are solvated in the MP or reside at the MP–aqueous interface. At low hexanol fraction, however, the lipids lose solubility and form a densely packed monolayer at the interface, thereby reducing the tension (Figure S3b). Similarly, evaporation of hexanol from a 10% hexanol fraction MP droplet during interfacial tension measurement results in a significant monolayer tension reduction until it reaches a plateau (see Note S3 and Figure S4). Such reduction in monolayer tension due to increased lipid packing is analogous to the Langmuir monolayer principle [41], with higher packing induced here by solvent depletion rather than mechanical compression. In Figure 4a, the γ_*MP* − *IP*_ is consistently higher than γ_*MP* − *OP*_ across all hexanol fractions ensuring that the OP provides a more favorable environment for the oil pocket than the IP, thereby promoting the oil pocket to reside outside cargo (see Note S4.2).

To evaluate the membrane (bilayer) tension under different hexanol fractions, the interaction between IP and OP droplets in MP solution within a microfluidic droplet interface bilayer (DIB) system was examined (Note S4.1). As shown in Figure 4b, three different hexanol fraction conditions of (i) 60%, (ii) 10%, and (iii) ≪10% were studied. The 60% hexanol fraction did not result in a bilayer formation at the interface (Figure 4b‐i), which corresponds to no dewetting of DEs (Figure S6‐i). Prolonged contact between two droplets under these conditions led to their coalescence. This condition corresponds to the burst of DEs at the onset of dewetting under high hexanol concentration (80% in Figure 2f). As bilayer formation is not energetically favorable in this case, contact between the inner and outer phase monolayers results in their coalescence and subsequent disruption of the DE. However, with a low hexanol fraction (10%) an interface bilayer was formed. In this case, if hexanol removal from the chip was prevented, the interface bilayer exhibited a low budding angle at equilibrium, akin to a low budding angle PDL (Figure 4b‐ii). If hexanol removal was permitted, the contact site between droplets continuously grew, and the budding angle (θ) increased until reaching an equilibrium morphology of high budding angle (Figure 4b‐iii). Figure 4c demonstrates such a continuous temporal increase in budding angle, parallel to that observed in PDL dewetting. This behavior reflects a significant reduction in the bilayer to monolayer tension ratio (γ_*b*_/γ_*m*_) during hexanol removal (see Note S4.1 for details), indicating that the bilayer tension decreases more rapidly than the monolayer tension. Using the measured monolayer tensions and the DIB geometries, we evaluated membrane tension and spreading parameters for the three dewetting morphologies (Figure 4e–g, Note S4 and Table S3). The results indicate that upon hexanol removal, monolayer and membrane tensions decrease markedly (Figure 4f), resulting in an increased outer phase spreading parameter (*S_OP_*) (Figure 4g). Since a higher *S_OP_* favors dewetting (Note S4.2), this change drives the morphological transition from low to high budding angle. Furthermore, this tension relaxation is rooted in the rearrangement of lipids during solvent removal. As hexanol is removed from the MP, lipids, by losing solubility in the oil pocket, change their distribution (Figure S7 and Figure 4d). We hypothesize that the desolvated lipids migrate toward the monolayers and the bilayer. The significant reduction in bilayer tension that we observed in γ_*b*_ Figure 4f is likely because of an increase in lipid density in the membrane since no change is applied to the inner and outer phase compositions throughout dewetting. Therefore, solvent removal induces lipid rearrangement and tension relaxation, hence drives the dewetting transition from DE to high budding angle PDL.

Poloxamer 188 is a triblock copolymer surfactant commonly used in liposome generation via solvent‐mediated dewetting method and has been introduced as the driving component for inducing dewetting in double emulsions [17, 42, 43, 44]. Here, we investigated the dewetting process in the absence of poloxamer 188 to identify its role in our dewetting system. Bilayer formation and dynamic changes in the budding angle under hexanol removal (Figure 4h) and reduction in γ_*MP* − *OP*_ (Figure 4i) also occur without poloxamer 188, following a progression similar to that observed in its presence. This signifies that solvent removal promotes dewetting regardless of the presence or absence of poloxamer 188 in our system. Therefore, solvent‐mediated dewetting can lead to liposome formation without the need for surfactant assistance. Notably, the non‐poloxamer DIB system exhibited a change in droplet size ratio over the incubation period, suggesting enhanced water permeability of the membrane under this condition.

### 2.3 PDL Morphology Post Solvent Removal: Achieving Complete Dewetting Is Non‐Spontaneous

**FIGURE 5:**
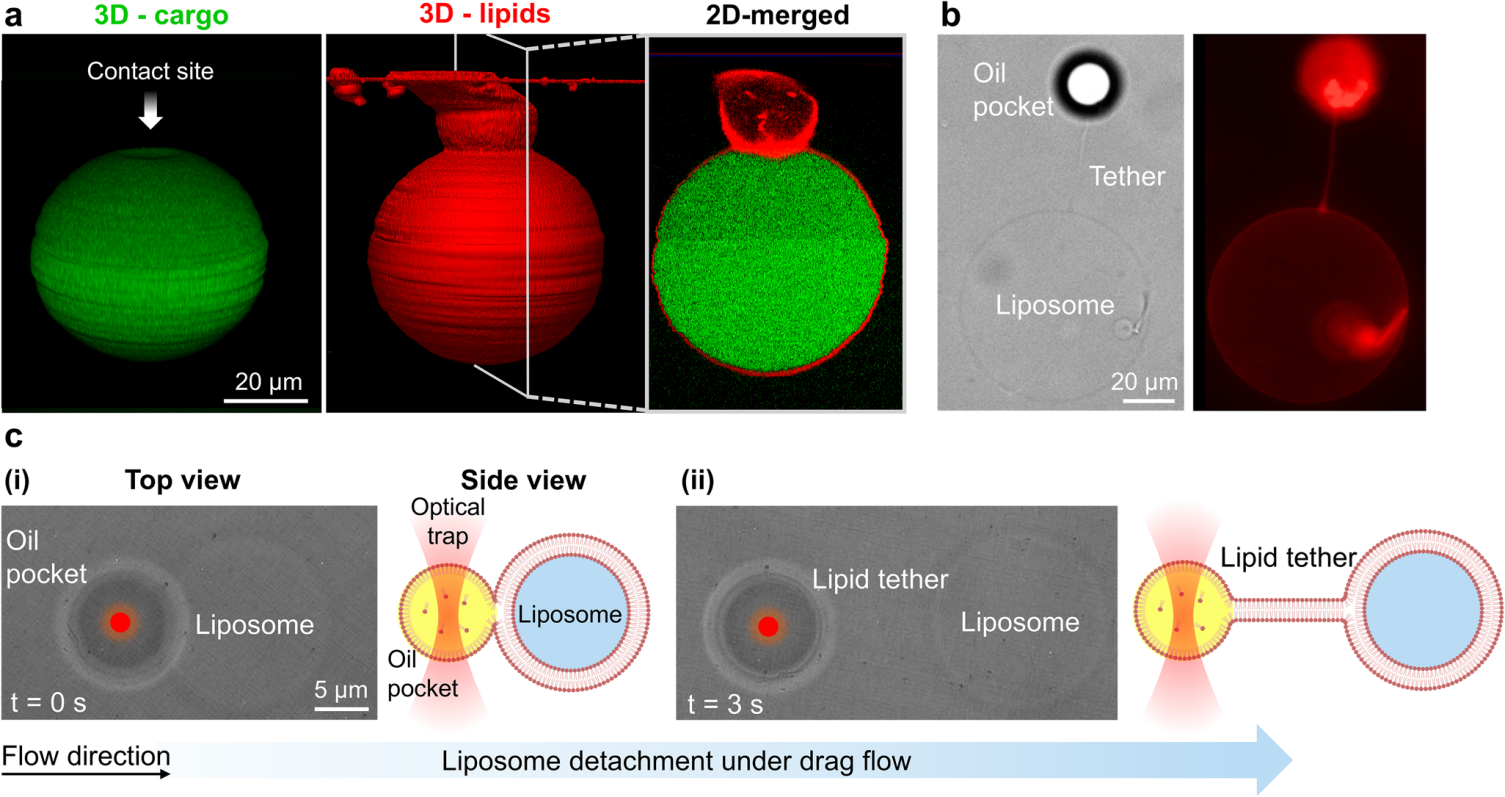
Oil pocket remains attached after solvent removal: Optical tweezer characterization. (a) 3D‐reconstructed confocal images of a high‐budding‐angle PDL and a cross‐section of the contact site between the liposome and the oil pocket after solvent removal. Lipids are shown in red; encapsulated cargo, in green. (b) Lipid tether formed between the oil pocket and the liposome following mechanical force, indicating physical continuity. (c) Optical tweezer experiment demonstrating pulling the liposome under a drag force. Microscopic image of the high‐budding‐angle PDL with optically trapped oil pocket (red spot) with the corresponding schematic of the side view. Continuous drag force exerted by the flow on the trapped PDL pulls against the liposome (i), leading to its separation and the formation of a lipid tether (ii).

Despite achieving high‐throughput and uniform formation of high budding angle PDLs in the PDMS gas‐permeable chamber, the oil pocket remains attached to the liposome, and complete budding is not achieved spontaneously (Movie S4). To further examine the non‐spontaneity of complete detachment, confocal z‐stack imaging was employed after solvent removal, where the PDL morphology was not changing anymore. As shown in Figure 5a and Movie S5, the liposomes are almost fully dewetted at this stage, but a contact site between the liposome and the lipid droplet is still present. Such a contact site has been observed in biological systems, particularly, it is shown that the spherical lipid droplets remained adhered to the endoplasmic reticulum membrane [30, 32, 45, 46, 47].

In some cases, as shown in Figure 5b, a prolonged connection between the oil pocket and the liposome in the form of a membrane lipid tether was observed. The residual adhesion between the liposome and the oil pocket was characterized by an optical tweezer (Note S5). Liposomes were difficult to trap due to low refractive index contrast with the medium, whereas the oil pocket in PDLs provided sufficient refractive index contrast, enabling strong optical trapping forces over 100 pN. This allowed optical manipulation of the entire structure using the trapped oil pocket as a handle.

To facilitate optical trapping of PDLs, the size of the generated DEs was reduced to approximately 10–20 µm in diameter. Fluid flow around the trapped PDL was then generated by controlled sample stage movement to exert drag forces. Under flow, the PDL aligned with the direction of movement (Figure 5c). The applied drag force pulled the liposome away from the oil pocket, forming a thin tether (Figure 5c‐ii). Upon cessation of flow, the oil pocket and liposome retracted into close proximity, indicating that this elastic strand maintains its contact in the absence of external stress. In most instances, lipids formed irregular connections extending over considerable distances, reaching tens of micrometers, before retracting, which prevented liposome detachment. Complete separation of the liposome from the oil pocket was achieved when tethers were notably shorter and thinner, with a drag force of 17–65 pN (Movie S6). Therefore, spontaneous detachment of the oil pocket from the liposome is prevented by tethering, requiring additional mechanical forces for complete dewetting. Notably, the longer the delay between PDL formation and the application of drag flow, the more difficult the separation becomes. We speculate that longer incubation time under slow hexanol evaporation rate allows lipids more time to reorganize and accumulate at the oil‐liposome interface, forming denser and more stable tethering that strengthens the adhesion.

We suggest that these lipid connections in the form of tubules or extended lipid tethers [48, 49] arise from pulling stress on the membrane. The tether formation observed in our system resembles cell tubulation [50] and vesiculation [33] and is likely driven by the low membrane tension after solvent removal, which facilitates strand growth [51]. Such tether has been also observed between the endoplasmic reticulum membrane and lipid droplet and is shown to facilitate the exchange of membrane proteins between these two compartments [52]. Figure S8 shows the application of drag flow to liposome at a low budding angle. Although the liposome undergoes deformation under the applied drag force, indicating the extent of the force, no tether formation or detachment is observed. This highlights the strong adhesion present at low budding angles and underscores the necessity of attaining a high budding angle morphology as a prerequisite for successful liposome detachment.

### 2.4 High‐Yield Generation of Liposomes with Complete Dewetting Transition

**FIGURE 6:**
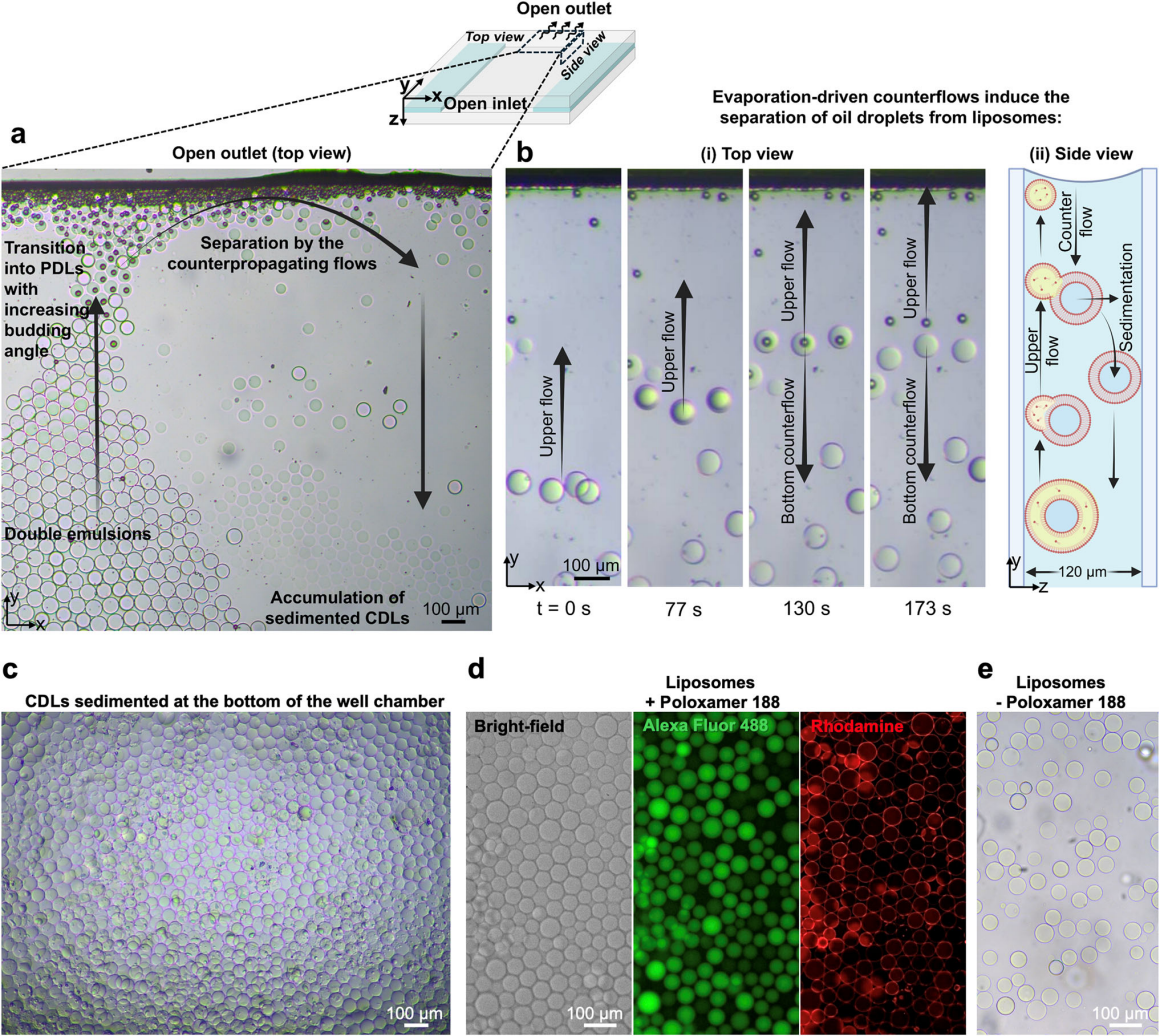
Evaporation‐driven counterflows enable high‐yield complete dewetting of liposomes. (a) Microscopy image at the outlet of the glass chamber, showing evaporation‐driven fluid flow within the chamber in conjunction with hexanol evaporation facilitate complete dewetting near the outlet. DEs moving toward the outlet initially transition into PDLs with low budding angles, which progressively increase as they approach the outlet. Separated CDLs then sediment and move backward, forming clusters of accumulated liposomes. (b) Time‐lapse images of DEs transition into low and high budding angle PDLs followed by complete detachment of liposomes (i), and a cross‐section schematic of the process (ii), demonstrating separation of liposome from the oil pocket near the outlet by counter‐propagating upper and counter flows. (c) Brightfield image of high‐yield CDLs accumulated at the bottom of the well. (d) Brightfield and fluorescence images demonstrating encapsulated Alexa Fluor 488 in cargo and Rhodamine in the liposome membrane. (e) Liposomes formed without poloxamer 188 using the same high‐yield formation approach as in (c) and (d).

In addition to using optical tweezers to achieve complete dewetting of the liposome from the oil pocket after solvent removal, we utilized an alternative evaporation‐driven dewetting mechanism to achieve a high yield of complete dewetting. When both the inlet and outlet of the glass chamber were left open, complete separation of liposomes from the oil pocket was observed near the outlet meniscus interface, driven by hexanol evaporation and shear flows induced by bulk outer phase evaporation. While hexanol diffusion from the oil shell into the outer phase chemically depleted the solvent, promoting the dewetting transition to a high budding angle morphology, concurrent evaporation of the outer phase generated internal flows, which created sufficient shear forces to enable complete dewetting (Figure 6a and Movie S7). The evaporation‐driven flow consisted of counter‐propagating streams, with the upper layer moving toward the outlet and the lower layer flowing backward. These counterflows pulled the oil droplet (dragged by the upper layer due to its lower density) and the liposome (dragged by the lower layer due to its high density) in opposite directions, facilitating their complete separation (Figure 6b, See Note S6 for estimation of detachment force). Detached oil droplets accumulated at the outlet, while the denser CDLs sedimented and moved backward within the chamber with the lower flow, forming clusters of hundreds of liposomes. Since the liposomes were freshly in a high budding angle state due to solvent depletion, only minimal shear was required for their complete separation.

Since solvent evaporation and mechanical force are essential mechanisms for effective dewetting to form liposomes, another method to achieve high‐yield liposome formation is shown by collecting DEs into a large open‐well chamber immediately after formation. The open chamber without a lid facilitated hexanol evaporation from DEs floating at the surface. Once a high‐budding‐angle morphology was achieved, the buoyancy force arising from the density difference between the liposomes and oil pocket relative to the continuous phase, with gentle flows generated by evaporation‐driven fluid circulation within the well, applied stress at the oil pocket‐membrane interface and promoted efficient CDL formation (See Note S6 for estimation of detachment force). Within 10–30 min, hundreds of liposomes sedimented at the bottom of the well (Figure 6c,d).

In addition, liposomes were formed in the absence of poloxamer 188 in the solution (Figure 6e), offering greater biocompatibility, as poloxamer may adversely affect membrane properties and protein functions in liposomes [53]. However, our storage analysis shows that liposomes with poloxamer 188 have higher long‐term stability (Figure S9). Furthermore, the unilamellarity of generated liposomes was confirmed by the successful incorporation of alpha‐hemolysin (α‐HL) transmembrane protein into the bilayer membrane (see Note S7 for details). Together, these findings demonstrate that a combined strategy of solvent removal and mechanical input enables the high‐yield formation of cell‐sized unilamellar liposomes.

## 3 Discussion

Understanding the dynamics governing morphological dewetting transitions is critical for (i) delivering a predictable approach to liposome generation, hence advancing synthetic cell engineering (ii) offering strategies for controlling dewetting morphologies, (iii) providing a mechanistic basis for experimental observations in dewetting‐based liposome generation; and (iv) determining the minimal requirements necessary to induce dewetting. Here, we identify solvent removal and mechanical force as key determinants governing the solvent‐mediated dewetting‐based assembly of liposomes. The depletion of lipid solvent (hexanol) from the oil pocket regulates the onset and progression of dewetting. Preventing solvent removal stalls dewetting; limited removal through semi‐permeable borders yields low budding angle PDLs with strong oil‐pocket affinity, while efficient removal at open borders drives a shift to high budding angle PDLs with weak affinity. This extends the classical wetting, partial dewetting, and full dewetting classification [17] by revealing distinct equilibrium intermediate stages within partial dewetting, modulated by the solvent fraction in the oil pocket, with potential implications in protocell research [12]. Uniform solvent evaporation in a gas‐permeable PDMS chamber leads to a spatially synchronized dewetting transition. In this case, a higher solvent removal rate, by using a thinner PDMS membrane, can bypass the low budding angle state and lead to a direct transition to high budding angle.

Our study delves into the origin of solvent‐mediated morphological transformation by demonstrating lipid rearrangement and analyzing interfacial tensions. As lipid solvent exits the oil pocket, pre‐dissolved lipids redistribute from the bulk to new affinity zones, such as interfaces. This leads to coordinated shifts in monolayer and, more prominently, membrane tensions, imposing a change in PDL morphology that satisfies the new interfacial tension balance. Therefore, a sequence of solvent depletion, lipid migration, and tension relaxation drives the dewetting morphology change.

Solvent removal is a necessary but not sufficient condition for complete dewetting. A high‐budding‐angle oil pocket remains adhered, indicating the presence of an energy barrier that impedes spontaneous detachment. Consequently, complete dewetting of liposomes requires mechanical force. The existence of such an energy barrier has been reported in various biological processes, including the budding of lipid droplet from cellular organelles [52], vesicle fission [54, 55, 56], and the division of membranous compartments [57, 58, 59, 60], where necking scission is mediated by specialized machineries such as scaffolding proteins, supporting the notion that oil pocket detachment is a non‐spontaneous process. The results observed in our system differs with the original description of dewetting‐based liposome formation as a fully spontaneous process [17]. There are contrasts regarding the spontaneity of complete dewetting in prior studies using solvent mediated dewetting method. Although the spontaneous oil droplet separation from liposome is stated in the literature [17], the non‐spontaneity of complete dewetting is evident in other studies following a similar solvent composition, in which either partially dewetted liposomes were used as the final product for synthetic cell research [27, 44, 61] or external force was proposed as a means to detach the oil pocket [62]. In addition, there are other dewetting studies using different solvents where PDLs are utilized as the dewetting outcome or mechanical tapping for complete detachment is recommended [20, 28, 63]. Together, these observations highlight that the complete dewetting in solvent‐mediated method is unlikely to be spontaneous. In our system, the residual adhesion and magnitude of the mechanical force required for complete dewetting are characterized. Using optical tweezers, we observed the formation of lipid tethers anchoring the oil pocket, which requires a detachment force on the scale of pN. Such a detachment force is introduced through evaporation‐driven flow and the buoyant force in high‐throughput conditions. Remarkably, similar dewetting behavior is observed in the absence of poloxamer, indicating that surfactants are not necessary for achieving solvent‐mediated dewetting.

The principles described here apply to morphological transitions observed in biological budding processes. While lipid solvent removal serves as the primary mechanism for modulating interfacial tensions and inducing budding in our synthetic system, living cells and vesicular organelles achieve a similar outcome through alternative strategies, such as lipid phase separation [32, 47, 64], localized protein accumulation [65], osmotic stress [66, 67], solute adsorption [55, 68], and cytoskeleton‐mediated contractility [35, 36]. Notably, the tension‐regulated increase of budding angle and the sustained association between oil pocket and lipid membrane observed in our system closely parallel the growth and fission of lipid droplets from endoplasmic reticulum membrane [32, 52, 69]. Thus, our findings provide biophysical insights into general principles underlying budding processes.

## 4 Conclusion

Here, we demonstrate that the dewetting of double emulsions into completely dewetted liposomes occurs via a series of distinct morphological transitions. The dynamics of progression from double emulsion (DE) to low and then high budding angle partially dewetted liposome (PDL) is controlled by solvent removal. Solvent removal causes increased lipid packing, a significant reduction in membrane tension, and triggers morphological transformation. However, despite predictions based on spreading parameters, complete dewetting is not spontaneous. We highlight the necessity of an external mechanical force to overcome the oil pocket's tethering to the liposome and quantify the range of the detachment force required for complete dewetting. Together, solvent removal and mechanical input constitute the solvent‐mediated dewetting framework, enabling high‐throughput generation of fully dewetted liposomes using a biocompatible hexanol‐paraffin oil solvent pair. Application of high‐resolution spectroscopic techniques to monitor solvent fraction and tension in the oil pocket in real time could be a future direction. Furthermore, identifying the molecular origin of the energy barrier to complete dewetting would facilitate establishing a more generic theoretical model predictive of wetting–dewetting states. These findings unveil the dewetting dynamics for liposome formation and offer an in vitro model for understanding tension‐regulated biological budding processes, particularly lipid droplet morphogenesis.

## 5 Experimental Section

### 5.1 Materials

Paraffin oil, glycerol (≥99% (GC)), 1‐hexanol (98%) (described as hexanol), *Staphylococcus aureus* alpha hemolysin protein (α‐HL), and Liss Rhod PE (1,2‐dioleoyl‐sn‐glycero‐3‐phosphoethanolamine‐N‐(lissamine rhodamine B sulfonyl (ammonium salt))) were purchased from Sigma Aldrich. Alexa Fluor 488 (A488, Invitrogen) and poloxamer 188 Non‐ionic surfactant (10% solution) were procured from Thermo Fisher, 2‐[methoxy(polyethyleneoxy)propyl] trimethoxysilane was purchased from Gelest. Polyethylene glycol (PEG, powder/flakes), *M*_W_ = 6000 gmol^−1^ was obtained from Alfa Aesar and DOPC lipid (1,2‐di‐(9Z‐octadecenoyl)‐sn‐glycero‐3‐phosphocholine) was purchased from Avanti Polar Lipids.

### 5.2 Hybrid PDMS/Glass Capillary Microfluidic Chip Fabrication

The microfluidic DE generation device used in this study is a hybrid PDMS/glass capillary system enabling prolonged formation without the need for selective surface treatments. This configuration also allows for straightforward capillary alignment and replacement, offering a robust platform for generating thin‐shell double emulsions across a broad range of sizes. The method has been described in detail in our previous paper [31] and a similar setup has been used for this work. Briefly, the PDMS replica features a two‐junction flow‐focusing design with a semi‐cylindrical outer channel and a conical tip configured to accommodate a glass capillary (outer diameter: 860 µm, inner diameter: 340 µm; Microcaps, Drummond Scientific). Two identical PDMS replicas, fabricated via standard soft lithography using a 3D‐printed mold, were aligned and bonded to form the PDMS part. The glass capillary was tapered using a capillary puller (PC‐10 Puller, Narishige International) and subsequently sanded to achieve a tip diameter of 80 µm. For extended capillary use, silanization was performed by filling the capillary with the silane solution and leaving it overnight. After thorough rinsing, the capillary was inserted into the outlet channel of the PDMS chip. Precise positioning of the capillary tip's horizontal and vertical alignment within the PDMS junction enabled high‐throughput formation of thin‐shell double emulsions with minimal oil droplet formation, operating in double‐dripping mode.

### 5.3 Double Emulsion Formation and Fabrication of Collection Chambers

DEs were generated using the following three phases. The inner phase (IP) consisted of an aqueous solution containing 8 wt% polyethylene glycol (PEG) and 5 µg mL^−^^1^ Alexa Fluor 488 (for cargo visualization). The middle phase (MP) was a 60:40 (vol%) mixture of hexanol and paraffin oil (unless otherwise specified), containing 8 mg mL^−^^1^ DOPC and 3 µg mL^−^^1^ Liss Rhod PE (for phospholipid visualization). The outer phase (OP) was an aqueous solution comprising 5 wt% glycerol and 1.5 wt% poloxamer 188 (unless otherwise specified).

To prepare the MP, the required volume of lipid stock solution (lipids dissolved in chloroform) was added to an amber glass vial to prevent lipid photooxidation. The chloroform was evaporated by vacuum drying leaving a lipid film, and the desired mixture of hexanol and paraffin oil was then added to the vial. The prepared solution was sonicated for 30 min in a room‐temperature bath.

A pressure pump (OB1 MK3, Elveflow) was used to introduce the freshly prepared phases into the hybrid microfluidic chip. The size and shell thickness of the resulting DEs were regulated by adjusting inlet pressures. Thin shell (1–5 µm) DEs with an average diameter of 60 µm and a throughput of approximately 600 DEs per second were produced. The DEs were flowed through the capillary and transferred via Tygon tubing (inner diameter: 500 µm) into either a glass or PDMS chamber. Each dewetting experiment conducted in the glass and PDMS chambers was repeated at least five times.

A glass chamber (length: 20 mm, width: 4 mm, height: 120 µm) was constructed from two glass coverslips (top and bottom walls) and double‐sided tape (side walls). DEs were collected into the chamber via capillary action, allowing single‐layer distribution for microscopic observation. Unless otherwise stated, both the inlet and outlet of the chamber were sealed with Vaseline (Vaseline Blue Seal).

A PDMS chamber was fabricated using a master mould created via a standard photolithography procedure. The PDMS device (length: 50 mm, width: 10 mm, height: 100 µm) was cast by curing a 10:1 PDMS curing agent mixture on the mould at 80°C for 2 h. Utilizing chambers with 50 µm height resulted in DE rupture, while 300 µm chambers caused multilayer stacking; therefore, a 100 µm height was found optimal for collecting 60 µm DEs. The cured PDMS was bonded to the glass coverslip after oxygen plasma treatment (Colibri, Gambetti).

To prepare a chamber with 1800 µm PDMS membrane, cured PDMS with a total thickness of 1900 µm was bonded to a glass coverslip. For a chamber with a 50 µm PDMS membrane, the cured PDMS block was bonded to a 50 µm PDMS sheet (limitless shielding). After bonding, the chamber was prefilled with the OP solution, then its protective layer was removed. The chamber was then placed membrane side down on a rigid surface to prevent deformation during DE collection. After DE filling, the membrane side was left open to allow solvent evaporation and observe dewetting.

The hexanol evaporation rate was calculated for PDMS chambers with 50 and 1800 µm thickness. The chambers were initially filled with pure hexanol, and the reduction in volume was monitored over time. Using the decrease in hexanol volume at specific time points and the dimensions of the chamber, the evaporation rates were calculated.

### 5.4 Visualization of the Dewetting Transition

DEs were observed immediately after collection using an inverted microscope (DM IL LED, Leica) equipped with a CMOS camera (Flexacam C1, Leica). During dewetting, the budding angle was examined using a 2D projection of side‐view images of PDLs and measured using ImageJ software. Confocal Z‐stacks and time‐series fluorescent images were acquired with a confocal laser scanning microscope (TCS SP8 with a DMI8 microscope, Leica), and fluorescence intensity plots were generated using Zen 3.5 acquisition software.

### 5.5 Interfacial Tension Measurement

Interfacial tension (IFT) was determined using the pendant drop method, with droplet shape analysis conducted via a tensiometer (Krüss DSA25). A J‐shaped needle (0.5 mm tip diameter) was used to generate droplets of the middle phase (MP) solution within a surrounding phase containing either the inner phase (IP) or outer phase (OP) solution. To ensure consistency and minimize alterations due to sample aging or hexanol evaporation, MP solutions were freshly prepared, sonicated for 30 min, and used immediately for each measurement. Once a droplet of sufficient volume was formed, measurements were conducted within the first 30 s and repeated for at least three droplets for each hexanol fraction (*N* = 3); the results were shown as means ± standard deviation (SD). Limiting measurements to newly formed droplets within a short time frame minimizes the risk of IFT alteration due to hexanol evaporation. Note S3 provides details on the measurement of IFT during hexanol evaporation.

### 5.6 Droplet Interface Bilayer Formation

DIBs were formed using a PDMS microfluidic T‐junction droplet formation chip with two junctions. Droplets of the IP, labeled with Alexa Fluor 488, and the OP were generated within the same chip (Figure S6a), while having freshly prepared MP as the continuous phase. In each experiment, the IP and OP solutions remained constant, while the MP solutions varied in hexanol fraction. The droplets were briefly incubated to allow monolayer formation before being brought into contact. Following this, bilayer formation and changes in the budding angle were monitored until equilibrium. To prevent solvent removal under condition (ii) in Figure 6b, the PDMS chip was placed inside an enclosed transparent container saturated with hexanol vapor. In addition, before use, the chip was soaked in a hexanol solution to saturate the PDMS gas solubility. The shape analysis for the calculation of membrane tension was done using ImageJ software. Detailed calculations for the evaluation of the membrane tension, spreading parameters, and their error estimation are provided in Note S4.

### 5.7 Optical Tweezer Setup and Measurements Procedure

Experiments were conducted in custom glass chambers assembled from two cover slips separated by double‐sided adhesive tape (height ∼80 µm). DEs had diameters of ∼15 µm, with oil droplets ∼5 µm and liposome sizes ranging from 8–15 µm. Due to differences in refractive index between the oil droplet (*n _paraffin oil_* ≈ 1.473, suggesting almost complete evaporation of hexanol at high budding angle morphology) and the outer aqueous phase (*n* ≈ 1.34), the optical trap selectively held the oil droplet, while the liposome remained untrapped. This allowed the oil droplet to be used as a stationary handle to trap the PDL. To minimize hydrodynamic drag from wall proximity, trapped PDLs located near the top surface were repositioned ∼20 µm downward. To quantify the detachment force, the sample stage was displaced laterally at a controlled velocity while maintaining trapping of the oil droplet. The stage velocity was incrementally increased until the liposome detached from the oil droplet. At the instant of detachment, the force exerted on the tether connecting the trapped oil droplet to the liposome equals the hydrodynamic drag acting on the liposome. Therefore, the drag force was calculated using the Stokes drag formula $F_{\mathrm{drag}}=6\pi \eta R_{\mathrm{lipo}\mathrm{some}}V_{\mathrm{crit}\mathrm{ical}}$, where η ≈ 1 mPa/s (pure water without glycerol), R is the liposome radius, and V is the velocity at the detachment event.

Near‐IR laser was chosen to operate in the biological transparency window, minimizing absorption and heating; prolonged trapping of oil pockets (>5 min) at maximum power 250 mW showed no effects on liposome membrane or oil‐pocket integrity.

### 5.8 High Throughput Formation of Completely Dewetted Liposomes

For evaporation‐induced liposome formation in a glass chamber, both the inlet and outlet were left open following DE collection. Evaporation occurred predominantly at the outlet, drawing the bulk solution in that direction. At the inlet, progressive evaporation caused the bulk solution boundary to recede toward the outlet. This bulk flow transports DEs in the direction of evaporation. As DEs approached the sample‐air interface near the outlet, dewetting was triggered, and PDLs transitioned from low to high budding angles followed by complete detachment of the oil pocket due to counterpropagating streams. The size ratio of liposome and oil pocket relative to the channel depth should be approximately below one‐half, allowing one to align with the upper flow while the other follows the counter‐stream flow. Experiments were conducted at laboratory temperature (21°C–23°C).

For bulk production of fully detached liposomes, Double emulsions (DEs) solution at a throughput of approximately 600 DEs per second was produced. 300 µL of this solution was collected directly from the outlet of the formation chip via a connecting tube into a well of a standard 12‐well polystyrene plate (Corning Costar 3513) within 5 min. Each collection well was prefilled with 1.5 mL of the outer aqueous phase to match the continuous phase used during DE generation. Upon collection, dewetting was observed near the air‐exposed surface of the well, driven by solvent removal from the oil pocket, followed by detachment of the oil pocket due to flow‐induced shear and buoyancy forces, forming completely dewetted liposomes (CDLs). The chamber was left open overnight to ensure further evaporation. The liposomes subsequently sedimented to the bottom of the well due to higher density (≈1.018 g.cm^−3^) compared to outer phase (≈1.015 g.cm^−3^), forming a compact single layer that could be easily visualized under a microscope. The resultant liposomes were collected by gentle pipetting from the bottom of the well for downstream analysis. For long‐term stability analysis, the well plate was covered and kept at 4°C. The number of stable liposomes in the corresponding wells was assessed in the following days.

### 5.9 Examination of Liposome Unilamellarity by Membrane Protein Insertion

To assess the unilamellarity of the generated liposomes, α‐HL stock solution was added to the IP solution to a final concentration of 10 µg/mL. A freshly prepared IP solution labeled with Alexa flour 488 fluorescent molecules was used for each experiment, and samples were imaged immediately after dewetting using a confocal microscope to analyze the subsequent release of molecules in case of pore formation. The fluorescent intensity time‐series images were acquired at 1.5‐minute intervals. The same procedure was followed for the control experiment without adding α‐HL in the IP solution. The α‐HL stock solution was prepared by dissolving 2 mg/mL α‐HL in an aqueous solution containing 10% (v/v) glycerol. The stock was stored at −20°C for short term use and at −80°C for long term storage.

## Author Contributions

M.B. and T.A. designed and performed the experiments, analyzed the data and wrote the manuscript. I.S.R and B.R. contributed to data validation and revised the manuscript. A.K. contributed in double emulsion formation setup and revised the manuscript. D.S. assisted in the analysis of membrane unilamellarity. C.E. designed the project, supervised the research and revised the manuscript.

## Conflicts of Interest

The authors declare no conflicts of interest.

## Supporting information




**Supporting File 1**: smll72627‐sup‐0001‐SuppMat.pdf.

**Supporting File 2**: smll72627‐sup‐0002‐MovieS1.mp4.

**Supporting File 3**: smll72627‐sup‐0003‐MovieS2.mp4.

**Supporting File 4**: smll72627‐sup‐0004‐MovieS3.mp4.

**Supporting File 5**: smll72627‐sup‐0005‐MovieS4.mp4.

**Supporting File 6**: smll72627‐sup‐0006‐MovieS5.mp4.

**Supporting File 7**: smll72627‐sup‐0007‐MovieS6.mp4.

**Supporting File 8**: smll72627‐sup‐0008‐MovieS7.mp4.

## Data Availability

The data that support the findings of this study are available in the supplementary material of this article.
