# Supplementary material for: Solvent‐Mediated Dewetting Principles for Cell‐Sized Liposome Formation
Source: Small. 2026 Feb 1;22(18):e12610. doi: 10.1002/smll.202512610 (PMC13014223; doi:10.1002/smll.202512610)
Supplement: Supplementary file 1 — Supporting File 1: smll72627‐sup‐0001‐SuppMat.pdf. [file SMLL-22-e12610-s004.pdf]

Supplementary information

Solvent-Mediated Dewetting Principles for Cell-Sized Liposome Formation

Mostafa Bakouei[1,†], *Tatiana Avsievich*[1,†], *Indraja Sundara Raju*[1], *David Stumpf*[1], *Ali Kalantarifard*[1], *Benny Ryplida*[1] & *Caglar Elbuken* *[1,2,3]

[1] Faculty of Biochemistry and Molecular Medicine, University of Oulu, Oulu, Finland

[2] Faculty of Medicine, University of Oulu, Oulu, Finland

[3] VTT Technical Research Centre of Finland, Oulu, Finland

* corresponding author

† these authors contributed equally

Table of contents

Description of Supplementary Movies

Supplementary Notes:
Note S1. Hexanol concentration calculation based on the partitioning coefficient
Note S2. The effect of solvent fraction on dewetting rate and formation quality
Note S3. Interfacial tension dynamics under hexanol fraction alteration
Note S4. Evaluation of membrane tension and spreading parameters using the DIB system
Note S4.1. Budding angle and membrane tension evaluation using the DIB system.
Note S4.2. Spreading parameters evaluation at different dewetting stages
Note S4.3. Error propagation in spreading parameter and membrane tension calculations.
Note S5. Optical tweezer setup details
Note S6. Estimation of detachment force in high-yield liposome generation systems
Note S7. Assessment of liposome membrane unilamellarity via membrane protein insertion

Supplementary Figures:
Figure S1. Low budding angle PDLs in the sealed glass chamber after 3 days.
Figure S2. High yield formation of high budding angle PDLs in gas-permeable PDMS chamber.
Figure S3. Monolayer tension as a function of hexanol fraction and lipid rearrangement
Figure S4. Evaluation of monolayer tension upon hexanol removal
Figure S5. Evaluation of budding angle and bilayer tension through droplet interface bilayer system
Figure S6. Equivalent morphological configurations in DIB and PDL systems
Figure S7. Lipid rearrangement during dewetting transition from low to high budding angle
Figure S8. Low budding angle PDL pulling using optical tweezers system
Figure S9. Long-term stability analysis of liposomes with and without poloxamer 188
Figure S10. Assessment of liposome unilamellarity using α-HL protein

Supplementary Tables:

Table S1. Hexanol and paraffin oil characteristics

Table S2. Effect of 1-hexanol fraction in the middle phase on dewetting.

Table S3. Evaluation of Monolayer tension, bilayer tension, and spreading parameters in the presence of poloxamer

Table S4. Evaluation of Monolayer tension, bilayer tension, and spreading parameters in the absence of poloxamer 188

Supplementary References

Description of Supplementary Movies:

Supplementary Movie 1
Description: Formation of double emulsions (DEs) using the hybrid PDMS/Glass capillary device. The microfluidic device operates in the double dripping mode, enabling high-yield formation (400 DEs/second) of thin-shell DEs.

Supplementary Movie 2
Description: Onset of dewetting and formation of partially dewetted liposomes (PDLs). Middle phase (MP) slowly accumulates to form an oil pocket as lipids self-assemble into a bilayer membrane.

Supplementary Movie 3
Description: A rapid and uniform dewetting transition within a gas permeable PDMS chamber.

Supplementary Movie 4
Description: Flow applied in PDMS gas-permeable chamber after achieving spatially uniform dewetting transition to high budding angle (1 h after DE formation), shows that the oil pocket is attached post solvent removal (no spontaneous dewetting). The PDLs are free-floating and not physically constrained (Size of DEs: 60-80 μm and depth of the chamber: 100 μm) hence no significant shear force is applied to them.

Supplementary Movie 5
Description: Confocal Z-stack video of a high budding angle PDL post solvent removal. This demonstrates an attached oil pocket with non-uniform lipid distribution. Lipids and cargo are labelled with Rhodamine and Alexa Fluor 488, respectively.

Supplementary Movie 6
Description: The oil pocket of PDL is optically trapped and held stationary. A drag force is applied to the liposome by moving the sample stage and inducing fluid flow.

Supplementary Movie 7
Description: Evaporation-driven complete dewetting in the glass chamber with open outlet, resulting in high yield formation of liposomes.

Note S1

Hexanol concentration calculation based on the partitioning coefficient. The partition coefficient of 1-hexanol between hexadecane and water can be used to approximate the partitioning of hexanol between the middle phase's other solvent (paraffin oil) and the aqueous phase (AP), since hexadecane is chemically similar to paraffin oil due to its highly non-polar, hydrocarbon-rich nature. From the experimental data reported in [2], hexadecane-water partition coefficient $ln\,K \approx 1.23$ which implies $K \approx 3.42$. Therefore, at equilibrium, the concentration of hexanol in hexadecane is approximately 3.4 times greater than in water. Applying the partitioning relationship:

$$K = \frac{[Hexanol]_{hexadecane}}{[Hexanol]_{water}} \approx \frac{[Hexanol]_{MP}}{[Hexanol]_{AP}} \quad \text{(S2)}$$

Having the initial concentration of hexanol in the MP according to 60% (v/v) hexanol fraction as:

$$[Hexanol]_{MP} = \frac{V_{hexanol} \times \rho_{hexanol}}{L} = \frac{600\ mL \times 0.82\ g/mL}{L} = 492\ g/L \quad \text{(S3)}$$

Therefore, the corresponding concentration of hexanol in the AP based on partitioning can be estimated as:

$$[Hexanol]_{AP} = \frac{[Hexanol]_{MP}}{K} = 144\ g/L \quad \text{(S4)}$$

This estimated aqueous concentration significantly exceeds the solubility limit of hexanol in water (~6 g/L), suggesting that in sealed chambers, the aqueous phase rapidly becomes saturated. As a result, further hexanol removal is suppressed in the absence of evaporation, explaining the arrested dewetting observed under sealed conditions. Enabling continuous hexanol removal through diffusion and evaporation via an open outlet or a gas-permeable PDMS membrane prevents outer aqueous phase saturation and sustains the progression of dewetting. We note that this calculation provides an approximation, and the exact concentration involves a more complex middle phase composition and the volume ratio of corresponding phases.

Note S2

The effect of solvent fraction on dewetting rate and formation quality. To further investigate the effect of lipid solvent (hexanol) on dewetting, we generated DEs with varying hexanol fractions in the MP (80, 60, 40, and 30 %) and assessed dewetting 5 minutes after their collection into glass chambers with an open outlet (Figure 2f). At the highest hexanol concentration (80%), the transition of DEs into PDLs was highly unstable, resulting in immediate rupture upon dewetting onset. Near the open outlet, only oil droplets from ruptured PDLs were observed. Reducing the hexanol fraction in the MP to 60% accelerated dewetting onset, enabling the formation of intact PDLs with progressing oil pocket budding at the outlet. At 40%, although the relative number of formed PDLs increased, DE formation was compromised, and artifacts such as multicompartment PDL were frequently observed. A further reduction to 30% hexanol led to a high number of CDLs and multicompartment PDLs immediately after chamber filling, suggesting rapid PDL formation after DE generation. Despite the high yield and fast transition into CDLs, the number of artifacts also increased. The formation of multicompartment PDLs is likely due to the wetting of capillary and transfer tubing walls at low hexanol fraction. Such wetting results in the formation of multicompartment DEs and coalescence of DEs where yields multicompartment PDLs after dewetting. While decreasing the hexanol fraction promoted faster dewetting, it also impaired DE formation, introducing undesirable features. Based on these observations, a hexanol concentration of 60% in the middle phase provides an optimal balance, ensuring robust DE formation and gradual dewetting while minimizing structural instability and artifacts (Table S2).

In contrast to the previous report[8] of a non-monotonic correlation between hexanol fraction and dewetting rate, we observe a consistent increase in dewetting rate with reducing hexanol fraction, underscoring the regulatory role of solvent removal. However, this introduces DE artifacts; whereas, a 60% starting fraction ensures robust formation.

Note S3

Interfacial tension dynamics under hexanol fraction alteration. To evaluate the interfacial tensions between MP and IP (γ_{MP-IP}) and also MP and OP (γ_{MP-OP}) after evaporation of a system with an initial 10% hexanol fraction (required for the measurement of bilayer tension in the scenario (iii) Figure 4b), the dynamic change in interfacial tension of a pendant droplet initially containing 10% hexanol was monitored over time (Figure S4). As the pendant droplet aged, hexanol from the middle phase gradually dissolved and diffused into the outer phase, akin to the hexanol removal process occurring in PDLs. As a result, the hexanol fraction decreased over time, leading to denser packing of lipids at the MP- OP and MP-IP interface and a subsequent reduction in monolayer tension (γ_{MP-IP}, γ_{MP-OP}) until it reaches a plateau. The final value of γ_{MP-OP} was significantly lower than γ_{MP-IP}. Measuring IFT at such low interfacial tensions using the pendant drop method is challenging, as the reduced interfacial tension increases the likelihood of droplet breakup, thereby limiting the volume of the pendant droplet that can be generated. To prevent droplet breakup while ensuring accuracy for OP:MP monolayer tension measurement, the evaluation began with a 0.4 µL droplet, which was gradually reduced to 0.1 µL at lower monolayer tensions. It was ensured that the IFT remained unchanged during the volume reduction. The Worthington number (Wo) shown in Eq. S5 indicates the accuracy of measurements in the pendant drop method, with a value close to 1 denoting a reliable measurement. Conversely, a Wo $\ll$ 1 suggests that the droplet volume is insufficient to accurately represent the shape of the droplet[3]. For the measurement with the lowest droplet volume (V_d = 0.1 µl) and $\gamma_{\mathrm{OP-MP}} = 0.15 \pm 0.05$, with a density difference ($\Delta\rho$ = 169 kg/m^3) and g = 9.81 m/s^2 the Wo value is 0.7 indicating that the measurement falls within the accurate range at this volume.

$$Wo = \frac{\Delta\rho g V_d}{\gamma \pi d_n} \quad \text{(S5)}$$

Note S4

Evaluation of membrane tension and spreading parameters using DIB system.

Note S4.1. Budding angle and membrane tension evaluation using DIB system. After microfluidic formation of the DIB system (Figure S5a), the angle between lipid monolayers of IP and OP droplets, defined as DIB budding angle (θ) can be derived from the geometrical parameters shown in Figure S5b using Eq. S6 where D_1 and D_2 are the diameters of the contacting droplets and d is the length of the contact line.

$$\theta = sin^{-1}\frac{d}{D_1} + sin^{-1}\frac{d}{D_2} \quad \text{(S6)}$$

Figure S5c, corresponding to the results presented in Figure 4c, illustrates the change in the DIB budding angle (θ) over time during hexanol removal. The experiment initially began with an MP solution containing a 10% hexanol fraction. Over time, due to gas removal through the PDMS microfluidic chip, the hexanol fraction in the MP solution gradually decreased. This reduction in hexanol fraction led to a progressive change in the DIB's budding angle, which reflects a decrease in the bilayer-to-monolayer tension ratio. To calculate the bilayer to monolayer tension ratio, an equivalent monolayer tension (γ_m) was defined for simplification. This γ_m represents a monolayer tension equivalent to $\gamma_{MP-OP}/\gamma_{MP-IP}$ in a symmetric DIB system with two droplets of identical content. Under this assumption, the bilayer to monolayer tension ratio γ_b/γ_m is related to the budding angle (θ) by Neumann's Triangle[4] shown in Eq. S7, and defined as:

$$\frac{\gamma_b}{\gamma_m} = 2\ \cos\frac{\theta}{2} \quad \text{(S7)}$$

Neumann's Triangle relation can be applied to measure the membrane (bilayer) tension. According to this relation, the surface tension balance must be satisfied for a given set of bilayer tension (γ_b) and monolayer

tensions (γ_{MP-OP}, γ_{MP-}) at a three-phase interface of a formed DIB (Figure 4b). The resulting relation is presented in Eq. S8, where as shown in Figure 4b(ii), α is the angle between the bilayer and IP monolayer and β is the angle between the bilayer and OP monolayer.

$$\gamma_b = (\gamma_{MP-IP} \cos\alpha + \gamma_{MP-OP} \cos\beta), \qquad \alpha, \beta \in [\pi/2, \pi] \tag{S8}$$

Therefore, by using the monolayer tension values measured via the pendant drop method and analyzing the morphology of the generated DIBs, membrane tension was determined. The calculated membrane tensions for three different scenarios shown in Figure 4b are presented in Table S3.

Note S4.2. Spreading parameters evaluation at different dewetting stages. Spreading parameters are a set of relations used to predict the wetting behavior of a three-phase liquid system[5]. In this study, we apply this framework to understand how hexanol removal influences the spreading parameters and how these changes impact the morphology of a PDL. For our system, the spreading parameters can be expressed as follows:

$$S_{IP} = \gamma_{MP-OP} - (\gamma_b + \gamma_{MP-IP}) \tag{S9}$$

$$S_{MP} = \gamma_b - (\gamma_{MP-IP} + \gamma_{MP-OP}) \tag{S10}$$

$$S_{OP} = \gamma_{MP-IP} - (\gamma_b + \gamma_{MP-OP}) \tag{S11}$$

In this framework, a negative spreading parameter for the inner phase ($S_{IP} < 0$) indicates that, if dewetting occurs, the oil pocket preferentially retracts toward the outer phase rather than becoming engulfed within the liposome cargo. A negative middle phase spreading parameter ($S_{MP} < 0$) indicates that the double emulsion undergoes dewetting, the resulted structure may exhibit a low or high budding angle PDL or a fully dewetted liposome depending on S_{OP}. A higher value of outer phase spreading parameter (S_{OP}) corresponds to an increased tendency for liposome dewetting therefore leading to a reduced contact area between oil pocket and liposome and a higher budding angle. When $S_{OP} > 0$ complete dewetting of liposome is expected. It is important to note that while the spreading parameters define the equilibrium state of the system and predict wetting and dewetting patterns within a given stage, they do not determine whether the transitions from DE to PDL or from PDL to CDL can occur spontaneously without external stimulation.

Based on the evaluation of monolayer and membrane tension under three different hexanol fraction configurations, the spreading parameters were calculated, as presented in Table 3. In the configuration with a 60% hexanol fraction (case (i)), the S_{MP} is positive, indicating that dewetting does not occur. This means even if the monolayers are forced into contact in a double emulsion, bilayer formation is not expected, and the double emulsion undergoes rupture. In the configuration where the MP contains a low hexanol fraction (10%) and evaporation is prevented (case (ii)), S_{MP} becomes negative, while S_{OP} increases. Hence, partial dewetting occurs, resulting in the formation of a PDL with a low budding angle. Finally, when evaporation is allowed, the resulting configuration (case (iii)) exhibits a significant increase in S_{OP}, further promoting dewetting and leading to the formation of an almost fully budded liposome. These findings indicate that hexanol removal alters monolayer and bilayer tensions in a manner that drives a continuous increase in S_{OP}, which in turn, enhances dewetting by reducing the contact area between the liposome and the oil pocket, and increasing the budding angle (Figure 4b, Table 3). Figure S6 illustrates the equivalent morphologies of the DIB and the DE/PDL systems under each of these three cases.

Our measurements shown in Figure 4a, indicate that γ_{MP} $< \gamma_{MP-I}$ across all tested hexanol fractions. Hence, regardless of the hexanol fraction, S_{IP} remains negative, ensuring that the oil pocket retreats toward the outer phase, facilitating outtake. This property provides an additional advantage for the choice of hexanol-paraffin oil mixture as middle phase for liposome formation in good-bad solvent method compared to the chloroform-hexane mixture, which offers a limited window for achieving a negative S_{IP}[6].

Note S4.3. Error propagation in spreading parameter and membrane tension calculations. To estimate the errors associated with bilayer tension and spreading parameters, a simplified error propagation equation was applied:

$$dS_{OP} = \sqrt{(\frac{\partial S_{op}}{\partial \alpha} d_{\alpha})^2 + (\frac{\partial S_{op}}{\partial \beta} d_{\beta})^2 + (\frac{\partial S_{op}}{\partial \gamma_{IP-MP}} d_{\gamma_{IP-MP}})^2 + (\frac{\partial S_{op}}{\partial \gamma_{OP-MP}} d_{\gamma_{OP-MP}})^2} \quad \text{(S12)}$$

By combining Eq. S8 and Eq. S11, the following expression is obtained:

$$S_{OP} = (1 + \cos\alpha)\gamma_{IP-MP} + (\cos\beta - 1)\gamma_{OP-MP} \quad \text{(S13)}$$

Where the partial derivatives are given by:

$$\frac{\partial S_{op}}{\partial \alpha} = -\partial\gamma_{IP-MP} \sin\alpha \quad \text{(S14)}$$

$$\frac{\partial S_{op}}{\partial \beta} = -\partial\gamma_{OP-MP} \sin\beta \quad \text{(S15)}$$

$$\frac{\partial S_{op}}{\partial \gamma_{IP-MP}} = (1 + \cos\alpha) \quad \text{(S16)}$$

$$\frac{\partial S_{op}}{\partial \gamma_{OP-MP}} = (\cos\beta - 1) \quad \text{(S17)}$$

As an example, for evaluation of dS_{OP} in scenario (iii), considering $\alpha = 110°, \beta = 100°, d_{\alpha} = d_{\beta} = 5°$, $\gamma_{IP-MP} = 0.5\text{mN/m}, \gamma_{OP} = 0.15\text{mN/m}$, $d_{\gamma_{IP-MP}} = d_{\gamma_{OP-MP}} = 0.05\text{mN/m}$, the calculated dS_{OP} is 0.08 mN/m.

A similar approach was employed to calculate the errors for γ_b, S_{IP} and S_{MP} presented in Table S3.

Note S5

Optical tweezer setup details. An in-house optical tweezers system was constructed based on a Zeiss Axiovert 100M inverted microscope operating in brightfield mode. The trapping laser (FPL852S, 852 nm, 350 mW, Thorlabs) was fiber-coupled and collimated using a FiberPort collimator (PAF2P-15B, Thorlabs), producing a beam diameter of 4 mm. The FiberPort was mounted on an XY slip plate positioner (SPT1, Thorlabs) for lateral alignment. Laser output power was finely controlled using a current and temperature controller (CLD1015, Thorlabs). The back aperture of the objective lens (UPlanSApo 100x/1.4 NA, oil immersion, Olympus) was slightly overfilled using a beam expander consisting of two lenses (AC254-60-B and AC254-150-B, Thorlabs). A dichroic mirror (FM01, Thorlabs; 95% reflection at 852 nm) was used to direct the IR beam into the optical path while transmitting visible light for imaging. The transmission of the objective at 852 nm was approximately 71-72%, providing an estimated power of up to 250 mW at the sample plane. Samples were observed using a CMOS camera (MV-D1024E-80-CL-12, Photonfocus) controlled via a custom MATLAB interface. The sample was positioned using a motorized scanning stage (SCAN IM 130x100-1mm, Marzhauser) with 0.01 µm resolution, operated via a TANGO 2D DT controller.

Note S6

Estimation of detachment force in high-yield liposome generation systems.

Estimated force in the evaporation-driven counterflow system. To estimate the detachment force of the liposome from the oil pocket, the velocities of the upper flow and the counterflow (Figure 6b) were determined by analyzing the corresponding experimental video. The velocity of the oil pocket after detachment was taken as the average velocity of the upper flow, while the velocity of the liposome after detachment was taken as the average velocity of counterflow.

For simplicity, the drag flow velocity was assumed to be equal to the average velocity of each flow stream, and the resulting drag forces on the liposome and the oil droplet were calculated using the Stokes drag formula:

$$F_{drag} = 6\pi\eta R_{particle} V_{flow} \tag{S18}$$

where η ≈ 1 mPa.s, R is the particle radius (R_{oil}~11 μm ,$R_{liposome}$~35 μm), and V is the fluid velocity. The velocity of the oil droplet (upper flow) was in the range of 1.5-2 μm/s, and the velocity of the liposome (counter flow) was in the range of 2.5-3 μm/s. Accordingly, the average force for the corresponding PDLs was estimated to be 0.3–0.4 pN for the oil droplet and 1.6–2 pN for the liposome. Since the drag force acting on these two particles are directed oppositely, both contribute to tether stretching and eventual detachment. Therefore, the estimated detachment force in this example lies in the range of 1.9–2.4 pN.

Estimated force in an open well chamber. For liposome detachment in well plate experiments, evaporation-driven flows may contribute in addition to buoyancy forces. However, due to the lack of visualization, only the buoyancy force was considered in the present analysis. The net buoyancy force acting on the liposome and the oil pocket can be estimated as

$$F_{net,\ buoyancy} = \Delta\rho V g \tag{S19}$$

where $\Delta\rho$ is the density difference between the fluid and the particle, V is the particle volume (R_{oil}~11 μm ,$R_{liposome}$~35 μm), and g is the gravitational acceleration.

Under the simplifying assumption that the oil pocket at the time of detachment consists solely of paraffin oil ($\rho_{parafin\ oil} = 850\ kg/m^3$ and with $\rho_{OP} = 1015\ kg/m^3$ the net buoyance force acting on the oil pocket is estimated to be 9 pN. For a liposome with $\rho_{IP} = 1018\ kg/m^3$, the net buoyancy force is estimated to be approximately 5.3 pN.

The buoyancy force drives the oil pocket upward and the liposome downward; thus, the total detachment force arising from buoyancy is given by the sum of these opposing forces, yielding an estimated value of approximately 14 pN.

In summary, the detachment forces estimated from three different experimental configurations (Optical tweezers, evaporation-driven counterflow, and open well chamber) fall within the lower piconewton range, spanning approximately 2–65 pN.

Note S7

Assessment of liposome membrane unilamellarity via membrane protein insertion. The unilamellarity of liposomes is a critical parameter for the protocell application of these vesicles. To evaluate the liposome's unilamellarity, we examined the insertion of the α-HL membrane protein. The α-HL self-assembles into the unilamellar membrane, enabling small fluorescent molecules (less than 2000 g mol−1) to diffuse through its ~2.6 nm pore, indicating a single lipid bilayer membrane[7]. As shown in Figure S10, following the addition of α-HL, a strong decay in the fluorescence intensity within the liposomes is observed over 15 minutes, due to the release of the encapsulated dye. In contrast, liposomes incubated without α-HL exhibited no significant fluorescence decay over the same period. These results confirm the unilamellarity of the generated liposomes, supporting their applicability in synthetic cell systems and drug development research.

Supplementary Figures

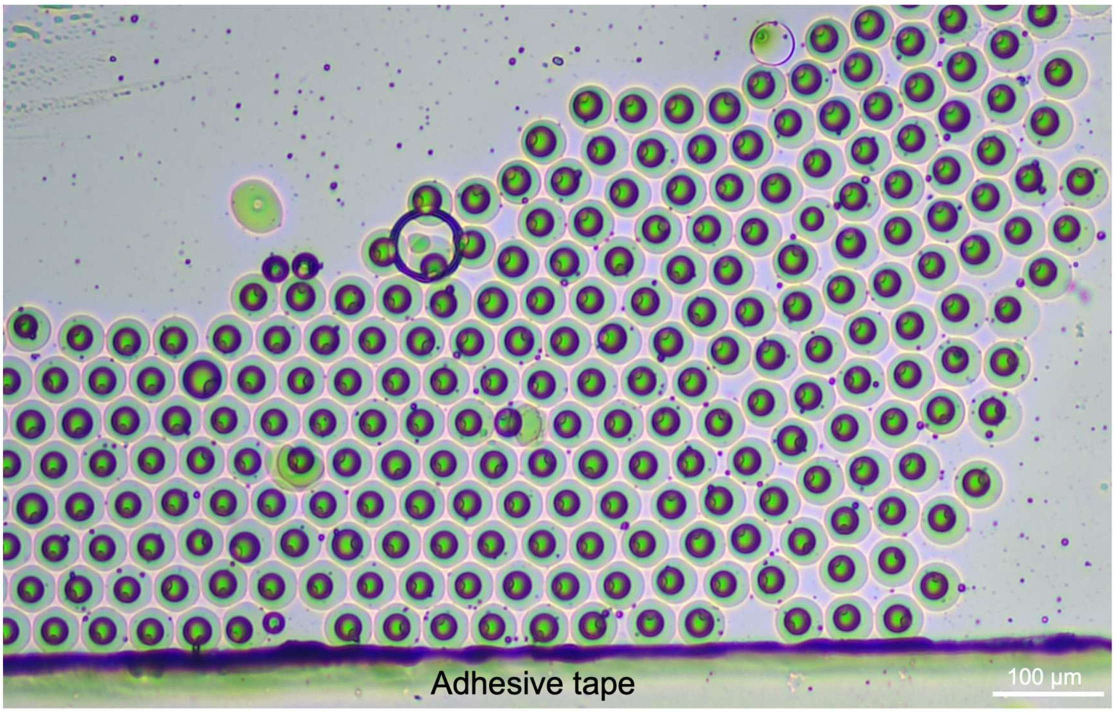

Figure S1. Low budding angle PDLs in the glass chamber in sealed chamber after 3 days.

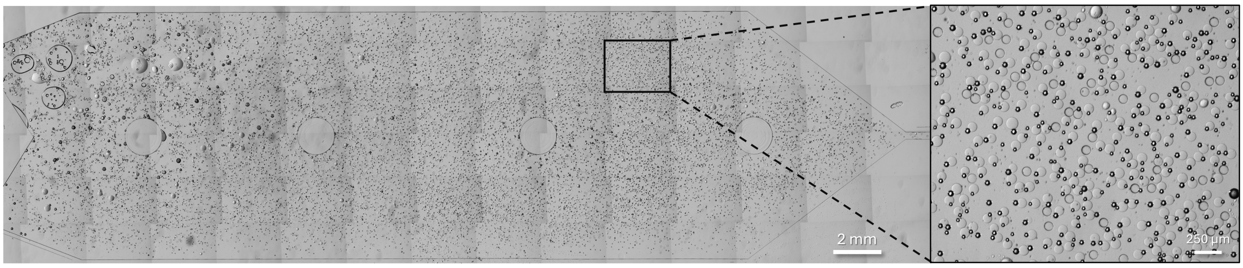

Figure S2. Tile image showing large scale formation of high budding angle PDLs in gas-permeable PDMS chamber.

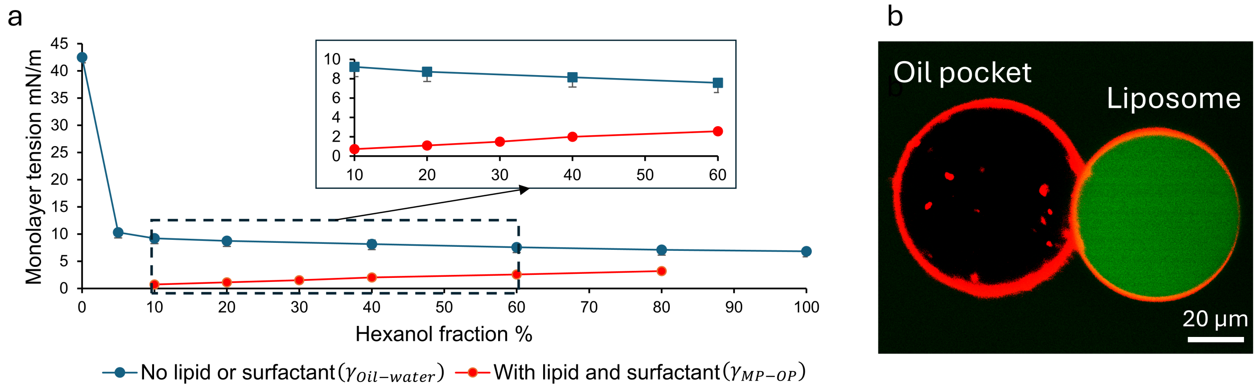

Figure S3. The influence of lipid, surfactant and hexanol fraction on monolayer tension of middle phase and lipid rearrangement in PDL after hexanol removal.
a*, Interfacial tension as a function of hexanol fraction in the presence and absence of lipid and surfactant. Oil includes hexanol: paraffin oil mixture without lipid.* ***b****, Confocal image of a high budding angle PDL after hexanol removal in the gas-permeable chamber. After hexanol removal, lipids lose their solubility in the oil and hence rearrange themselves by either forming aggregates or adhering to the monolayer interface. Lipids are labeled with rhodamine (red), and liposome cargo is labeled with Alexa Fluor 488 (green). Error bars indicate the mean±SD (n=3)*

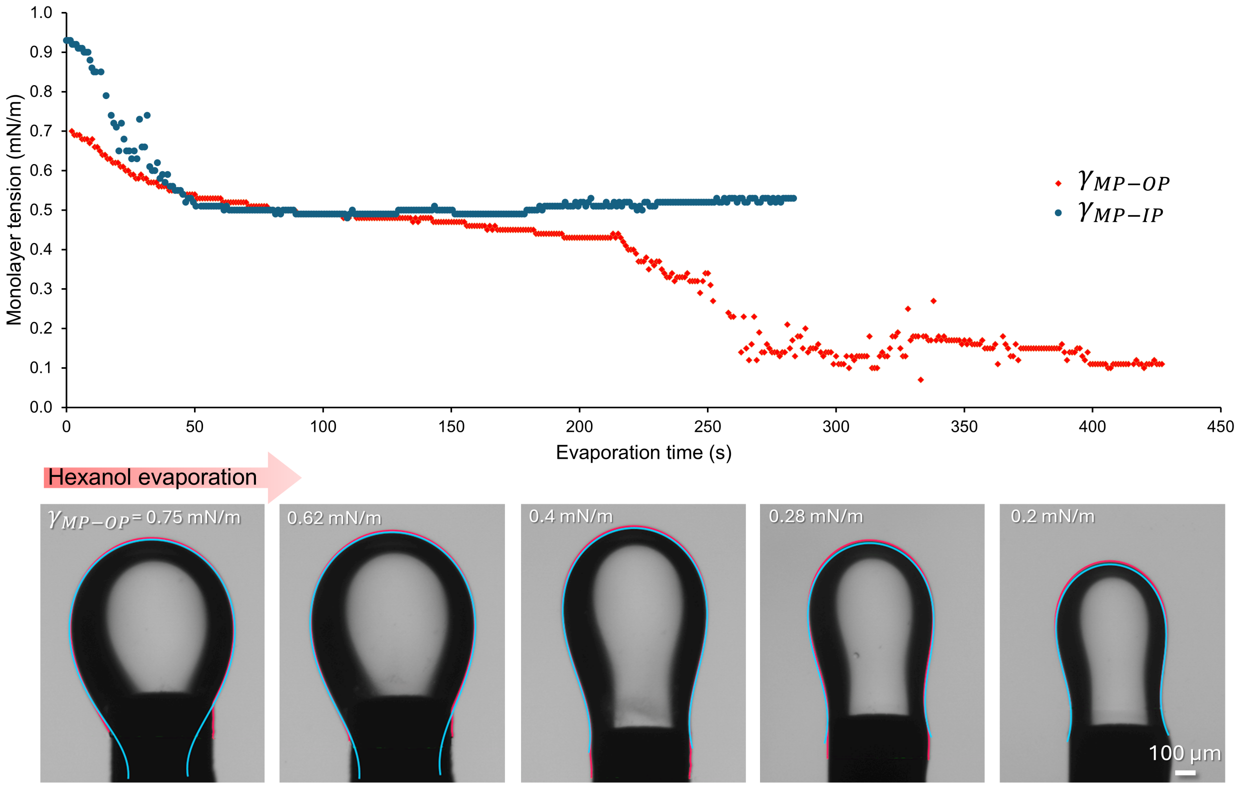

Figure S4. Evaluation of monolayer tension upon hexanol removal using MP solution for the pendant drop with an initial 10% hexanol fraction. *The sequential images at the bottom represent the corresponding MP pendant drop shape alteration in the OP solution as a result of hexanol evaporation.*

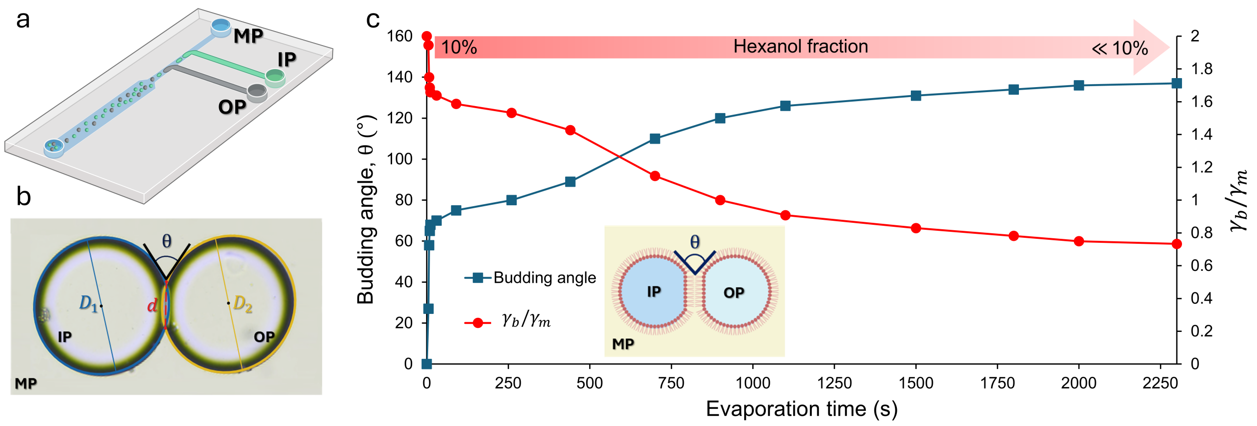

Figure S5. Evaluation of budding angle and bilayer tension through droplet interface bilayer system
a*, Schematic illustrating the microfluidic droplet formation setup used for DIBs formation.* ***b****, Diagram representing the geometrical factors used in a DIB system to measure the budding angle by Eq. S6.* ***c****, Dynamic change of the DIBs budding angle (θ) and the bilayer to monolayer tension ratio during hexanol evaporation over time. The MP solution initially contained a 10% hexanol fraction. The inset schematic represents the budding angle (θ), the angle between two monolayers of lipid in a formed DIB.*

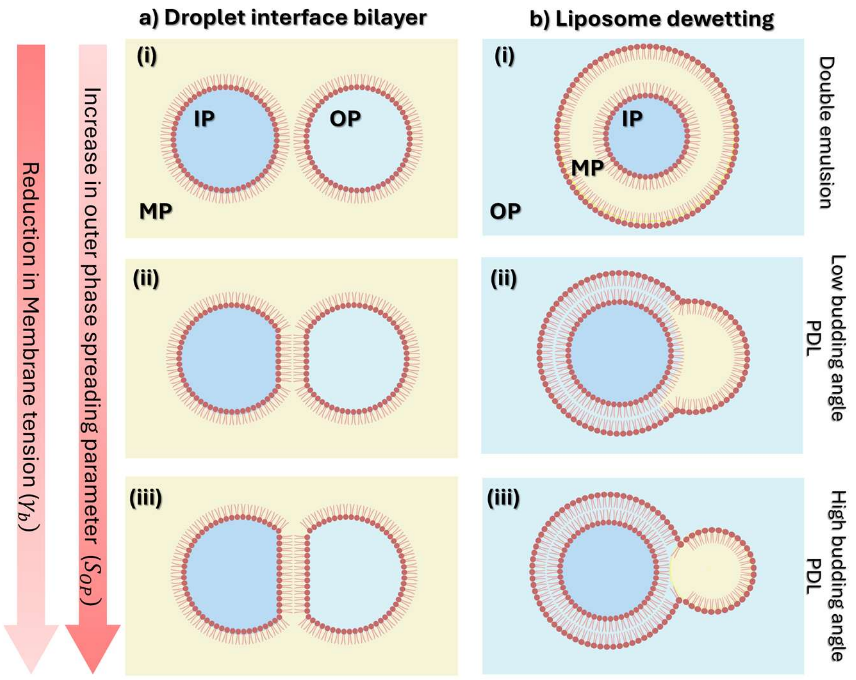

Figure S6. Equivalent morphological transformation in DIB and PDL systems upon hexanol removal. *Reduction in tension and increase in* S_{OP} *lead to higher budding angle and increased bilayer surface area in both systems.*

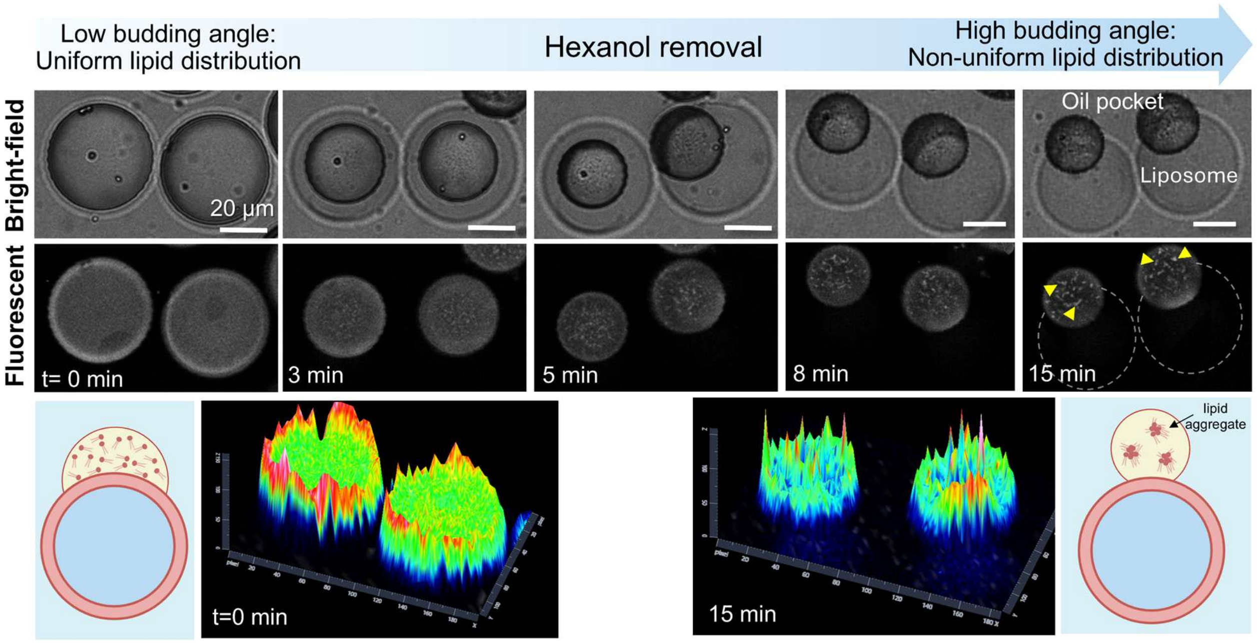

Figure S7. Lipid rearrangement during dewetting transition from low to high budding angle. *Time-lapse bright-field and fluorescence images of lipid rearrangement wihin the oil pocket during the transition of PDLs from low to high budding angle in a PDMS chamber. Rhodamine-labeled lipids in the oil phase were used to visualize lipid dynamics during solvent removal. At the initial time point (t=0 min), lipids are uniformly distributed within the oil pocket; As dewetting proceeds, the fluorescence signal becomes increasingly non-uniform, with localized lipid aggregated (yellow arrows) and presence of lipid-depleted regions. Corresponding 2.5D fluorescence intensity maps at 0 and 15 min reveal lipid clustering in oil pockets of high budding angle PDLs, where intensity Peaks represent lipid aggregates, and low-intensity regions indicate lipid-depleted zones. Schematics illustrate the lipid distribution in the oil pocket of low and high budding angle PDLs.*

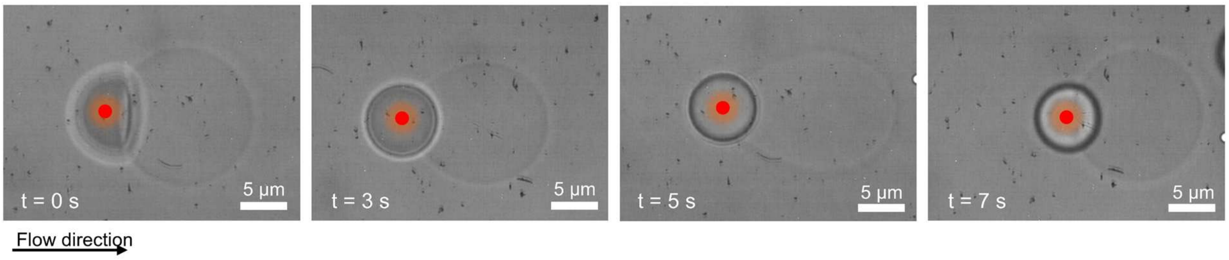

Figure S8. Low budding angle PDL pulling using optical tweezers system. *strong adhesion prevents oil pocket detachment at low budding angle PDL under drag flow. Continuous drag force exerted on liposome while the oil pocket is held in an optical trap. At t=0 s and t=7 s the flow is stopped.*

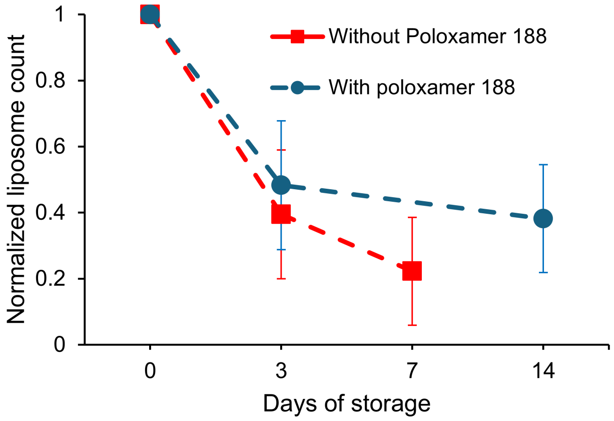

Figure S9. Long-term stability analysis of liposomes with and without poloxamer 188. *Following collection in the open well chamber, liposome stability was assessed over a period of two weeks. During this time, the liposomes were stored at 4 °C. Unstable liposome either form smaller vesicles or lipid aggregates; therefore, liposomes smaller than DE size were excluded from the analysis. For each data point, at least 1,000 freshly collected liposomes were counted. Error bars represent the mean±SD (n=3). The choice to use poloxamer 188 depends on the intended application. Applications requiring higher membrane mimicry favor surfactant-free liposome formation, whereas long-term stability necessitates the use of a surfactant.*

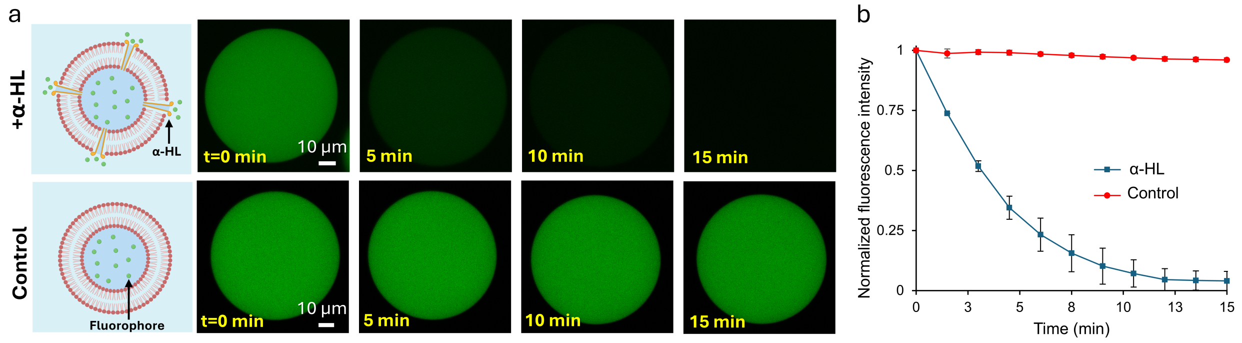

Figure S10. Assessment of liposome unilamellarity using α-HL protein.
a*, Confocal time-series images showing the release of fluorophore molecules (Alexa Fluor 488) from the liposome cargo within 15 minutes in the presence of α-HL. In the absence of α-HL (control), no significant loss in fluorescence intensity over the same period is observed.* ***b****, Corresponding normalized fluorescence intensity over time for liposomes with α-HL (blue square) and without α-HL (red circle). Error bars indicate the mean±SD (n=3).*

Supplementary Tables

Table S1*. Solubility in water and volatility values for hexanol and paraffin oil, and the corresponding quality of solvent for DOPC.*

Component	*Solubility in water (at 20°C), g/L*	*Volatility in air (Boiling Point)*	*Solubility of DOPC*
Hexanol	**~5.9**	**Moderate (Boiling Point: 157°C)**	**Good**
Paraffin Oil	**<0.001**	**Low (Boiling Point: >300°C)**	**Very poor**

Table S2. *Effect of 1-hexanol fraction in the middle phase on dewetting.*

	Hexanol fraction, %	Paraffin oil fraction, %	Formation	Dewetting	
Better formation ↑	80	20	Good formation	Very slow dewetting, PDLs burst immediately after formation	Faster dewetting ↓
	60	40	Good formation	Moderate dewetting, stable PDLs	
	40	60	Poor formation, multicompartment DEs	Faster dewetting	
	30	70	Bad formation, merging, wetting, many multicompartment DEs	Dewetting happened very soon after DE formation, hundreds of liposomes formed upon transfer to the chamber	

Table S3. *Evaluation of Monolayer tension, bilayer tension, and spreading parameters under three different hexanol fraction cases corresponding to Fig. 4b: (i) 60%, (ii) 10%, and (iii) ≪10% hexanol fraction, in the presence of 1.5 wt% poloxamer 188 in the OP solution.*

Hexanol fraction, %	***Monolayer tension (mN/m)***		***Bilayer tension (mN/m)***	***Spreading parameters (mN/m)***			***Equivalent PDL form***
	γ_{IP-MP}	γ_{OP-MP}	γ_b	S_{IP}	S_{MP}	S_{OP}	
(i) 60	3.5±0.47	2.6±0.19	≥6.1±0.5	≤-7.0±0.9	≥0	≤-5.2±0.4	No dewetting
(ii) 10 (No solvent removal)	1±0.07	0.75±0.05	1.6±0.09	-1.85±0.1	-0.15±0.03	-1.35±0.1	Low budding angle
(iii) ≪10 (After solvent removal)	0.5±0.05	0.15±0.05	0.2±0.05	-0.58±0.09	-0.42±0.07	0.15±0.08	High budding angle

Table S4. *Evaluation of Monolayer tension, bilayer tension, and spreading parameters under three different hexanol fraction cases of: (i) 60%, (ii) 10%, and (iii) ≪10% hexanol fraction, in the absence of poloxamer 188 in the OP solution.*

Hexanol fraction, %	***Monolayer tension (mN/m)***		***Bilayer tension (mN/m)***	***Spreading parameters (mN/m)***			***Equivalent PDL form***
	γ_{IP-MP}	γ_{OP-MP}	γ_b	S_{IP}	S_{MP}	S_{OP}	
(i) 60	3.5±0.47	3.5±0.1	≥7±0.5	≤-7.0±0.9	≥0	≤-7±0.4	No dewetting
(ii) 10 (No solvent removal)	1±0.07	0.8±0.05	1.7±0.09	-1.9±0.1	-0.1±0.03	-1.5±0.1	Low budding angle
(iii) ≪10 (After solvent removal)	0.5±0.05	0.25±0.05	0.22±0.05	-0.47±0.09	-0.53±0.07	0.03±0.08	High budding angle

Supplementary References

1. Bakouei, M. *et al.* Facile and versatile PDMS-glass capillary double emulsion formation device coupled with rapid purification toward microfluidic giant liposome generation. *Microsyst Nanoeng* **10**, 1–11 (2024).

2. Schantz, M. M. & Martire, D. E. Determination of hydrocarbon-water partition coefficients from chromatographic data and based on solution thermodynamics and theory. *J Chromatogr A* **391**, (1987).

3. McHale, G., Afify, N., Armstrong, S., Wells, G. G. & Ledesma-Aguilar, R. The Liquid Young's Law on SLIPS: Liquid-Liquid Interfacial Tensions and Zisman Plots. *Langmuir* **38**, 10032–10042 (2022).

4. Hui, C. Y. & Jagota, A. Planar equilibrium shapes of a liquid drop on a membrane. *Soft Matter* **11**, 8960–8967 (2015).

5. Ho, C. S., Kim, J. W. & Weitz, D. A. Microfluidic fabrication of monodisperse biocompatible and biodegradable polymersomes with controlled permeability. *J Am Chem Soc* **130**, 9543–9549 (2008).

6. Deng, N. N., Yelleswarapu, M. & Huck, W. T. S. Monodisperse Uni- and Multicompartment Liposomes. *J Am Chem Soc* **138**, 7584–7591 (2016).

7. Langzhou Song *et al.* Structure of Staphylococcal α-Hemolysin, a Heptameric Transmembrane Pore. *43. J. T. Nickels and J. R. Broach, Genes Dev* **270**, 2025 (1995).

8. Chien, P. J., Shih, Y. L., Cheng, C. T. & Tu, H. L. Chip assisted formation of phase-separated liposomes for reconstituting spatial protein-lipid interactions. *Lab Chip* **22**, 2540–2548 (2022).
